# Microfluidically Aligned Collagen to Maintain the Phenotype of Tenocytes In Vitro

**DOI:** 10.1002/adhm.202303672

**Published:** 2023-12-08

**Authors:** Francesca Giacomini, David Baião Barata, Hoon Suk Rho, Zeinab Tahmasebi Birgani, Clemens van Blitterswijk, Stefan Giselbrecht, Roman Truckenmüller, Pamela Habibović

**Affiliations:** ^1^ Department of Instructive Biomaterials Engineering MERLN Institute for Technology‐Inspired Regenerative Medicine Maastricht University Universiteitssingel 40 Maastricht 6229 ER The Netherlands; ^2^ Instituto de Medicina Molecular Faculdade de Medicina Universidade de Lisboa Avenida Professor Egas Moniz Lisbon 1649‐028 Portugal

**Keywords:** cell morphology/shape, collagen alignment, microfluidics, phenotype maintenance, tenocytes

## Abstract

Tendon is a highly organized tissue that transmits forces between muscle and bone. The architecture of the extracellular matrix of tendon, predominantly from collagen type I, is important for maintaining tenocyte phenotype and function. Therefore, in repair and regeneration of damaged and diseased tendon tissue, it is crucial to restore the aligned arrangement of the collagen type I fibers of the original matrix. To this end, a novel, user‐friendly microfluidic piggyback platform is developed allowing the controlled patterned formation and alignment of collagen fibers simply on the bottom of culture dishes. Rat tenocytes cultured on the micropatterns of aligned fibrous collagen exhibit a more elongated morphology. The cells also show an increased expression of tenogenic markers at the gene and protein level compared to tenocytes cultured on tissue culture plastic or non‐fibrillar collagen coatings. Moreover, using imprinted polystyrene replicas of aligned collagen fibers, this work shows that the fibrillar structure of collagen per se affects the tenocyte morphology, whereas the biochemical nature of collagen plays a prominent role in the expression of tenogenic markers. Beyond the controlled provision of aligned collagen, the microfluidic platform can aid in developing more physiologically relevant in vitro models of tendon and its regeneration.

## Graphical abstract

A user‐friendly microfluidic piggyback platform is developed allowing the controlled patterned formation and alignment of collagen fibers simply on the bottom of culture dishes. Rat tenocytes cultured on the micropatterns of aligned fibrous collagen exhibit a more elongated morphology and show an increased expression of tenogenic markers compared to tenocytes on tissue culture plastic or non‐fibrillar collagen coatings.

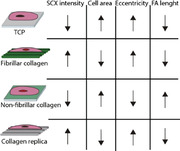

## 1 Introduction

Tendons make joint movement possible by transferring forces generated by muscle contractions to bones. Structurally, tendon tissue has a highly organized, hierarchical extracellular matrix (ECM), predominantly consisting of aligned type I collagen fibers, which in turn are formed by the aggregation of aligned collagen fibrils.^[^ ^1^ ^]^ The main cell type populating tendons are tenocytes, which are specialized fibroblasts. They are responsible for the secretion of ECM proteins and the maintenance of tissue homeostasis, regulating the level of ECM turnover.^[^ ^2^ ^]^ Tenocytes are mechanosensitive cells,^[^ ^3^ ^]^ and their phenotypical and functional characteristics rely among others on the collagen organization within the tendon ECM to act as a microtopographical cue.^[^ ^4^ ^]^

Once damaged because of trauma or inflammation, tendons have a limited healing capacity as result of a poor vascularization and low cell number.^[^ ^5^ ^]^ In injured tendons, the original parallel alignment of ECM shifts into a more isotropic organization in which collagen fibers exhibit a smaller diameter. Under such conditions, tenocytes lose their elongated shape, thereby undergoing a change in their orientation from head‐to‐tail to side‐by‐side alignment, and exhibit a decrease in the expression of tendon‐related genes, such as scleraxis (*Scx*) and tenomodulin (*Tnmd*).^[^ ^6^ ^]^ Surgical interventions are not fully effective in reconstructing damaged tendon tissue.^[^ ^7^ ^]^ Therefore, regenerative medicine (RM) approaches are under development with the aim to recover the original tendon tissue architecture and function.^[^ ^8^ ^]^

An important challenge in these approaches is the culture of tenocytes in vitro in an adequately physiological microenvironment. When cultured on conventional tissue culture plastic (TCP), the microtopographical guidance of the natural, highly organized ECM is lacking. This often leads to the loss of the elongated shape of tenocytes and a decrease in the expression of *Scx*,^[^ ^9^ ^]^ collagen‐I (*Col‐I*), decorin (*Dcn*), and tenascin‐C (*Tnc*).^[^ ^5^, ^10^ ^]^ Such a phenomenon, also known as “dedifferentiation,” is not only limited to cells of the tenogenic lineage but represents a general problem in the field of tissue engineering (TE) and RM.^[^ ^11^ ^]^

Different strategies have been pursued to modify cell culture substrates such that the tenogenic characteristics of cells are maintained, taking advantage of the biomechanical cues induced by surface topographies. For example, to identify the optimal surface topography establish a tenogenic niche in vitro, a chip‐type library of microtopographies in polystyrene (PS) was screened for topographical features, such as “feature size” and “pattern area,” related to *Scx* expression.^[^ ^9^ ^]^ In other studies, microgrooved SU‐8 epoxy resin‐glass,^[^ ^12^ ^]^ poly(lactic‐*co*‐glycolic acid),^[^ ^13^ ^]^ and fibronectin‐coated silicon substrates were shown to force cells into an elongated shape partly enhancing the expression of tendon‐related genes.^[^ ^14^ ^]^ While these studies demonstrate that surface microtopography can be a useful tool for inducing and maintaining the tenogenic phenotype, they were limited to materials that are clearly different from the natural ECM of tendon. Other studies focused on the effects of collagen‐based biomaterials. For example, Kishore et al. observed an increased expression of tenogenic markers and genes in mesenchymal stem/stromal cells (MSCs) on electrochemically aligned collagen (“ELAC”) threads in comparison to randomly oriented collagen threads.^[^ ^15^ ^]^ Similarly, MSCs on (in‐plane aligned) collagen sheets obtained with electrochemical compaction (“ECOM”) showed, compared to uncompacted sheets, a more spread and elongated morphology and a higher proliferation.^[^ ^16^ ^]^ Anisotropic collagen matrices were also produced by applying magnetic fields,^[^ ^8^, ^17^ ^]^ though not for applications in tendon repair and regeneration. In addition, electrospun scaffolds from a poly(l‐lactic acid) (PLLA) and collagen blend have been shown to induce morphological modifications of Hs27 human foreskin fibroblasts, which appeared elongated and aligned with nanofiber bundles of the scaffolds.^[^ ^18^ ^]^ These studies underline the impact of aligned matrices on stimulating the tenogenic phenotype of cells by manipulating their shape.

Besides biofabrication technologies, also microfluidic systems^[^ ^19^ ^]^ have been explored to generate micropatterns of aligned collagen fibers. For example, based on hydrodynamic focusing, a microfluidic device has been used for the self‐assembly of collagen‐I gels into fibrils and fibers in defined flow fields and at controlled viscosity and pH.^[^ ^20^ ^]^ Collagen fiber alignment has been observed in microfluidic channels with a diameter below 100 µm.^[^ ^21^ ^]^ Microfluidically aligned collagen fibrils/fibers have been used to study changes in corneal keratocyte morphology and orientation^[^ ^22^ ^]^ as well as in neuronal cell morphology, neurite/axon outgrowth and orientation.^[^ ^23^ ^]^ Microfluidically aligned collagen type I structures were also shown to maintain and allow multilineage differentiation of MSCs toward the osteogenic, adipogenic, and chondrogenic lineages and to enhance myotube organization/assembly and length of C2C12 mouse myoblast cells.^[^ ^24^ ^]^ Together, these studies have demonstrated that microfluidic alignment and consequently topographical patterning of collagen can be used to steer cell behavior, including fate commitment.

In the present study, we developed a novel, easy to use piggyback microfluidic platform for the controlled formation and alignment of collagen‐I fibers with different extents of orientation simply on the bottom of culture dishes. We then investigated whether patterns of microfluidically aligned collagen fibers contribute to the phenotypic maintenance of mature tenocytes in vitro, and also sustain the expression of tenocyte‐related markers in MSCs. This was achieved by varying the shape and spacing of hexagonally or parallel arranged micropillars integrated in the flow chambers of microfluidic devices, which in turn enforced different velocity fields in the microchannel networks or parallel microchannels between the micropillars. From device fabrication, to collagen fiber alignment and to cell readouts, the platform is easy to set up and run. In addition, using imprinted PS replicas of aligned collagen fibers, we decoupled the fibrillar structure of the ECM protein from its biochemical properties.

## 2 Results

### 2.1 Design, Fabrication, and Dimensional and Flow Characterization of the Microfluidic Platform

**Figure 1:**
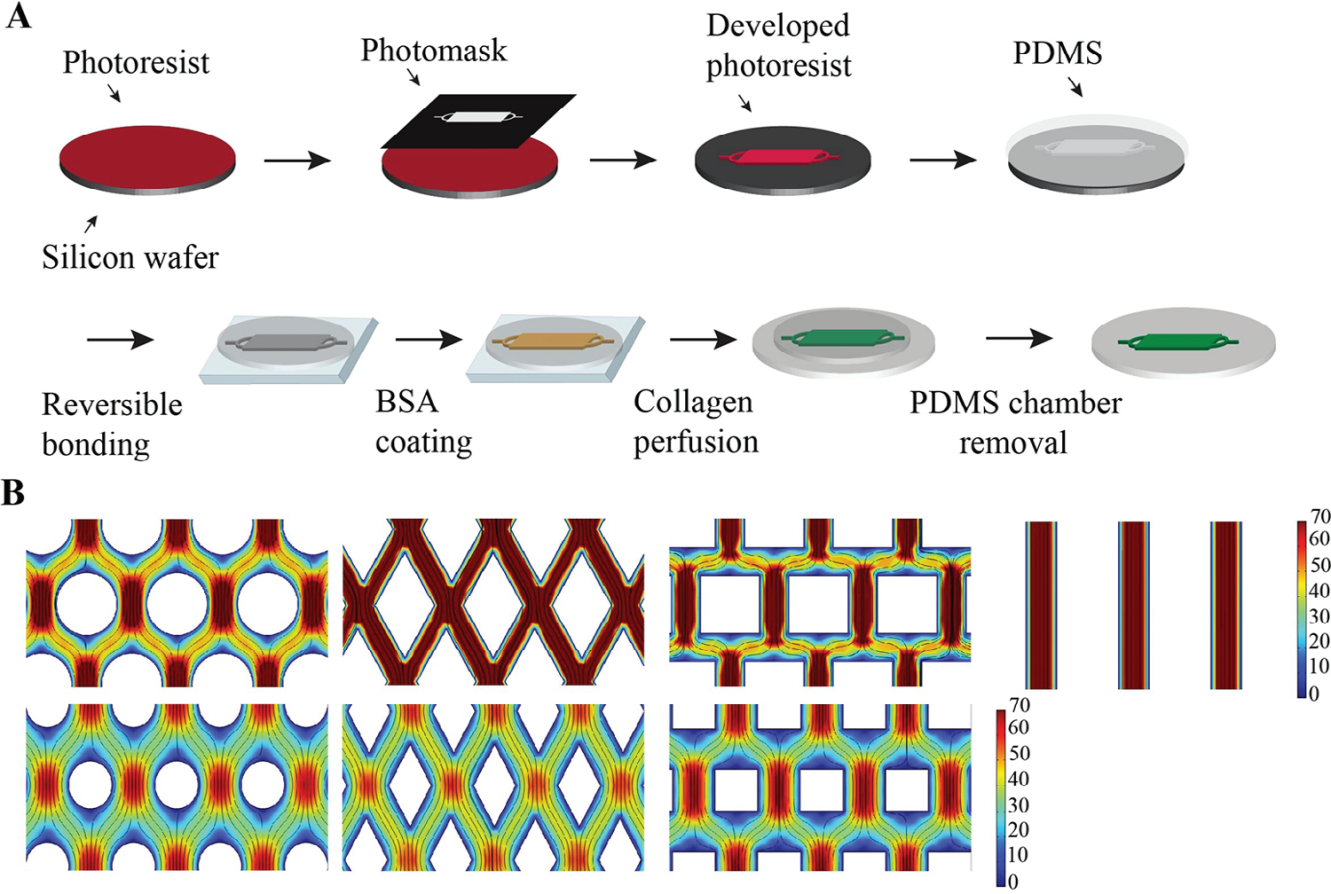
Concept, design, and characterization of the microfluidic platform. A) Schematic of the fabrication process of the microfluidic devices (top row) and collagen micropatterns (bottom row). B) Velocity heat maps and flow lines (dark blue), displaying the local flow directions, from CFD simulations of the laminar flow through a central section of the pillared array of the microfluidic devices with different shapes of the pillars—circle, rhombus, square, and line (left to right)—and spacing between them—50 µm (top row) and 100 µm (bottom row). The two color legends at the right represent the local flow velocities in mm s^−1^.

For the platform, different microfluidic devices were designed. They comprised flow chambers with various integrated hexagonally and parallel arranged arrays of micropillars (with the parallel ones in the form of aligned straight walls) running from the chamber floors to their ceilings. The micropillars had different shapes and spacing between them (Table S1, Supporting Information). The resulting designs were named after their micropillar shape and (minimum) pillar wall‐to‐pillar wall spacing/clearance in micrometers as “Circle 50,” “Circle 100,” “Rhombus 50,” “Rhombus 100,” “Square 50,” “Square 100,” and “Line.” A device without pillars and referred to as “Blank” was also designed, based on the assumption that it leads to a low alignment of collagen fibers. Using a photolithographically fabricated SU‐8 mold, the top parts of the microfluidic devices were fabricated by polydimethylsiloxane (PDMS) casting (**Figure** **1**A; top row). The flow chambers had a base area of 1.5 mm × 4 mm and a height of 35 µm. The quality of the lithographic structures was assessed by confocal laser profilometry, showing uniform widths and height, closely resembling the original computer designs (Figure S1, Supporting Information).

Computational fluid dynamics (CFD) simulations were performed to predict the laminar flow (concerning local velocity and direction) for the different micropillar array designs (Figure S2, Supporting Information; showing, as an example, the designs Circle 50 and 100). As introduced in the previous section, microflows can support the alignment of collagen fibers.^[^ ^19^ ^]^ Expectedly, the CFD simulations predicted higher (average) flow velocities for a micropillar spacing of 50 µm compared to that of 100 µm (Figure 1B; top and bottom row, respectively). This can be assumed to be reflected in higher collagen fiber alignment for the smaller interpillar spacing (which includes the Line design).

### 2.2 Microfluidic Preparation and Characterization of Collagen Micropatterns

**Figure 2:**
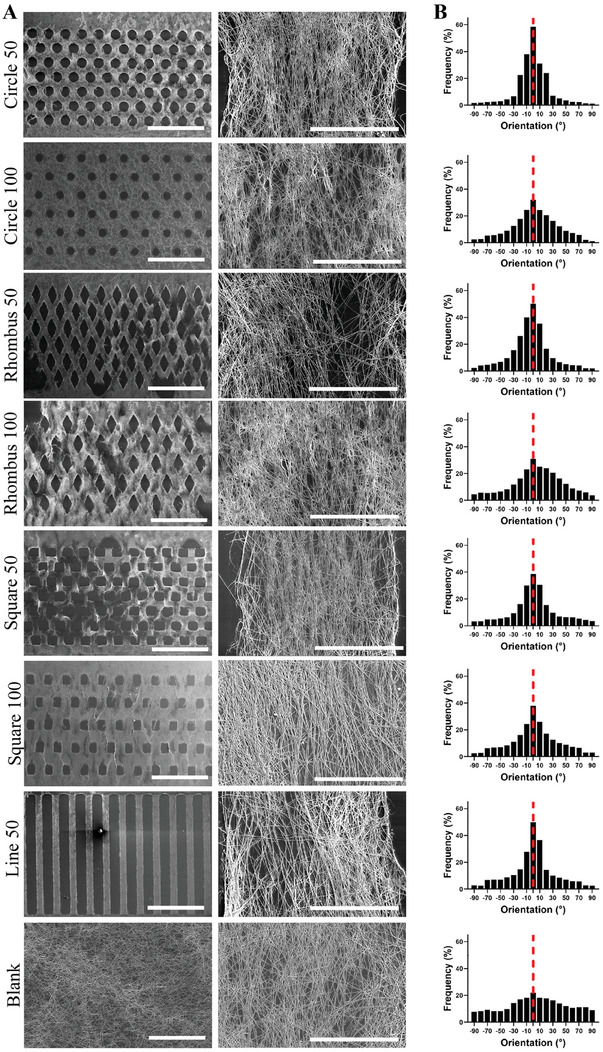
Formation of collagen fibers obtained with the different microfluidic devices. A) SEM images of collagen fibers formed in the microchannels by micropillars of different shape and spacing. Scale bars in left and right column represent 1 mm and 50 µm, respectively. B) Quantitative analysis of collagen fiber alignment. Histograms represent for the different micropillar array designs and the Blank design, as in each case indicated on the left side under (A), the relative frequency (*y*‐axis) of total fibers within each angle range versus the fiber orientation angle (*x*‐axis), with 0° (red dashed line) set as parallel to the left edge of the image. *N* = 3.

Following bovine serum albumin (BSA) passivation of the PDMS top parts of the microfluidic devices, collagen micropatterns were obtained on glass substrates by flowing a stabilized collagen‐I solution through the micropillar arrays (Figure 1A; bottom row) followed by an incubation step at 37 °C. Scanning electron microscopy (SEM) images of collagen micropatterns (**Figure** **2**A) confirmed the formation of collagen fibers. The characteristic D‐banding of 65 nm was observed on high‐magnification SEM images independent of the micropattern type (Figure S3A, Supporting Information), which is a confirmation that the collagen assembled in the correct, periodically structured fibrillar form.^[^ ^25^ ^]^ The average fiber diameter inside the bundles was around 150 nm (Figure S3B, Supporting Information).

Image‐based quantification of the collagen fibers’ orientation showed a strong dependence on the spacing of the micropillar array inside the chamber of the microfluidic device (Figure 2B). The fiber orientation distributions were used to calculate an orientation index, namely as the percentage of fibers oriented at 0 ± 5° (Table S2, Supporting Information). Fiber alignment was evident in the Circle 50, Rhombus 50, Square 50, and Line micropatterns, with a percentage of fibers oriented around 0° of 60%, 50%, 55%, and 50%, respectively. Fiber alignment was less pronounced for a micropillar spacing of 100 µm; orientation indices of 32%, 31%, and 38% were determined for the designs Circle 100, Rhombus 100, and Square 100, respectively. In the Blank device, a fiber alignment of 22% was determined. Taken together, these results show that collagen fibers were formed and aligned within our microfluidic devices, and that the micropatterns yielding the best fiber alignment were the ones with the smaller, 50 µm spacing, which is what one would have also anticipated considering the CFD simulations.

A rheological characterization of the collagen solution when exposed to a temperature ramp assumedly not so dissimilar from the above‐mentioned incubation step shows the increase of the collagen's shear modulus and viscosity with temperature and over time (Figure S4, Supporting Information). This is an indication of a rapid collagen polymerization and might have contributed to maintain the collagen fiber order.

### 2.3 Effects of Collagen Micropatterns on the Shape of Tenocytes and Their Nuclei

**Figure 3:**
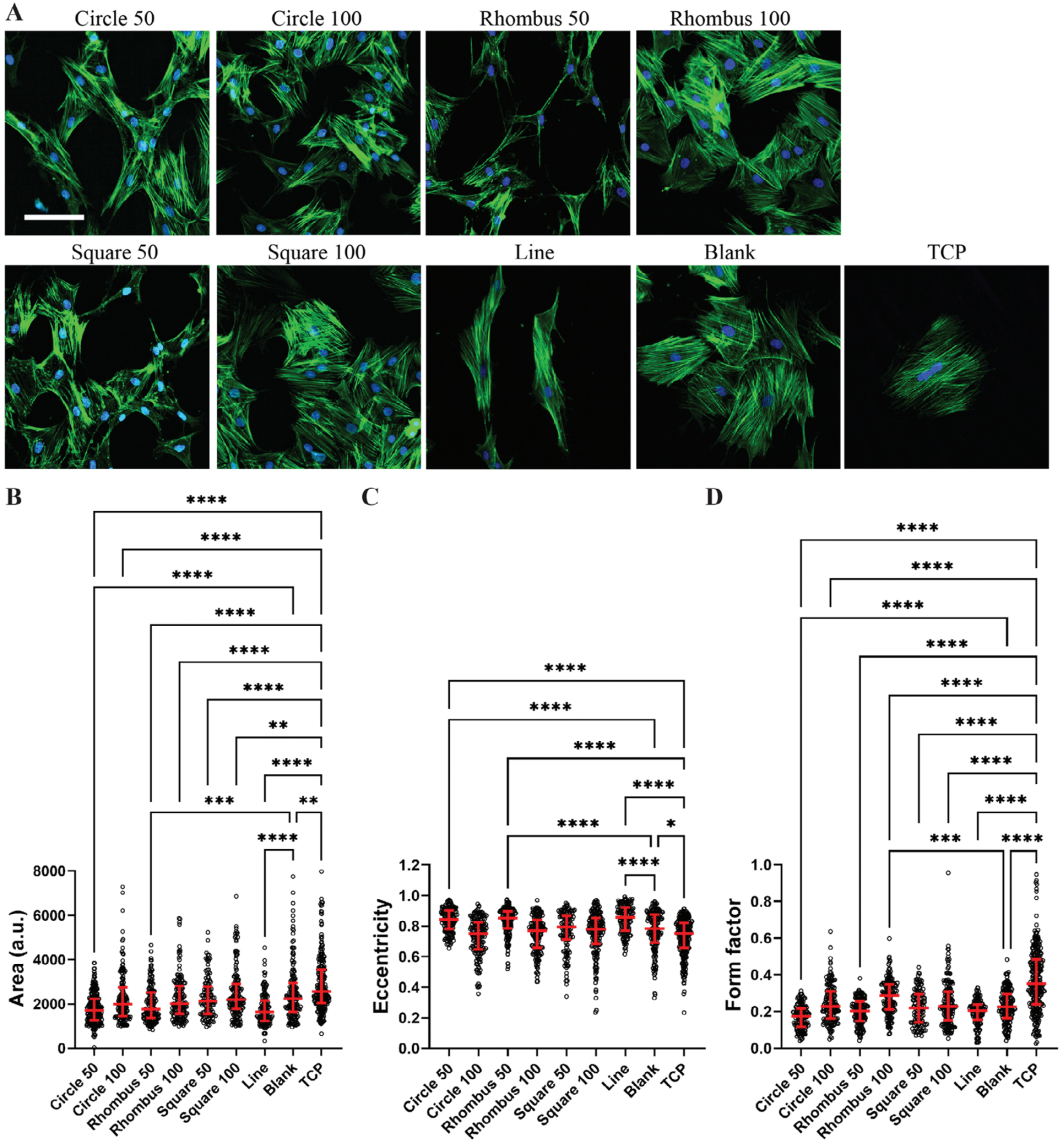
Characterization of the morphology of tenocytes cultured on collagen fibers for 3 days. A) Representative immunocytochemistry images of rat tenocytes cultured on fibrous collagen micropatterns formed in microfluidic devices with different micropillar arrays and without (Blank) and on TCP. Tenocytes were stained with DAPI to visualize cell nuclei (blue) and phalloidin to visualize F‐actin (green). Scale bar represents 50 µm and applies to all images. B–D) Quantitative analysis of cell shape descriptors, including cell area (B), eccentricity (C), and form factor (D). Median values are represented by horizontal lines and interquartile ranges by error bars. *p < 0.05, **p < 0.01, ***p < 0.001, ****p < 0.0001. *N* = 3.

To determine the ability of the different collagen micropatterns to support the maintenance of the mature tenogenic phenotype, the morphology of the cells cultured on the micropatterns after 1 and 3 days of culture was analyzed. It is important to mention that in our study we used cells from an already comparatively high and therefore more demanding passage number 5, while studies typically use lower and “easier” passage numbers 3 or 4.^[^ ^9^, ^13^, ^26^ ^]^ On day 1, we observed that tenocytes cultured on collagen fibers obtained with Rhombus 50 and Line devices displayed a significantly smaller cell area when compared with TCP controls (Figure S5A,C, Supporting Information). In addition, the eccentricity (i.e., the ratio of the distance between the foci of an ellipse and its major axis length, which can take values between 0, for a circle, and 1) of cells cultured on collagen micropatterns from Circle 50 and Square 50 devices was significantly higher than that of cells cultured on collagen from Blank devices (Figure S5A,C, Supporting Information). The eccentricity of cells is a measure for their elongation. Also, the form factor of cells was significantly smaller for all micropatterns from devices with a 50 µm interpillar spacing (including Line devices) in comparison with TCP (Figure S5A,C, Supporting Information). The form factor of the cells was calculated as 4 × π × cell‐area : cell‐perimeter^2^ (which can take values between 1, for a circle, and 0) and is a measure for the cells’ circularity. Tenocytes grown on Circle 50, Rhombus 50 and Line collagen micropatterns for 3 days showed a significant reduction in area compared to tenocytes grown on either Blank collagen substrates or TCP (**Figure** **3**A,B). The eccentricity of tenocytes was significantly higher upon culture on collagen micropatterns from Circle 50, Rhombus 50 and Line devices compared to Blank devices (Figure 3A,C). The form factor of tenocytes cultured on collagen fibers obtained with Circle 50 and Rhombus 100 devices was significantly smaller compared to Blank devices and TCP (Figure 3A,D). Regarding the morphology of the tenocytes’ nuclei after 3 days of culture, we observed a significant reduction in the nuclear area of tenocytes cultured on collagen patterns compared to those on TCP controls (Figure S6, Supporting Information). Similarly, nuclear eccentricity significantly increased in cells cultured on collagen patterns compared to cells on TCP controls. Additionally, we measured a significant rise in nuclear eccentricity in Rhombus 50 and Line devices compared to Blank devices. The nuclear aspect ratio, which indicates cellular elongation, exhibited a significant increase in cells cultured on collagen patterns, with the exception of Rhombus 100 devices, when compared to cells on TCP controls. The aspect ratio was also higher in Rhombus 50, Square 50, and Line devices compared to Blank devices. Taken together, these observations demonstrated that collagen micropatterns, obtained using our microfluidic platform and remaining stable over the culture duration (Figure S7, Supporting Information), can affect the cytoskeletal organization of tenocytes. Cells with a smaller cell area and more elongated morphology were in the first instance obtained on collagen micropatterns resulting from the micropillar arrays with a spacing between the pillars of 50 µm. These cells resembled the morphology of early‐passage primary tenocytes (Figure 3A; see, for example, Circle 50 image), as reported in previous work.^[^ ^4^, ^9^ ^]^

### 2.4 Analysis of SCX Expression and TNC Production by Tenocytes on Collagen Micropatterns

**Figure 4:**
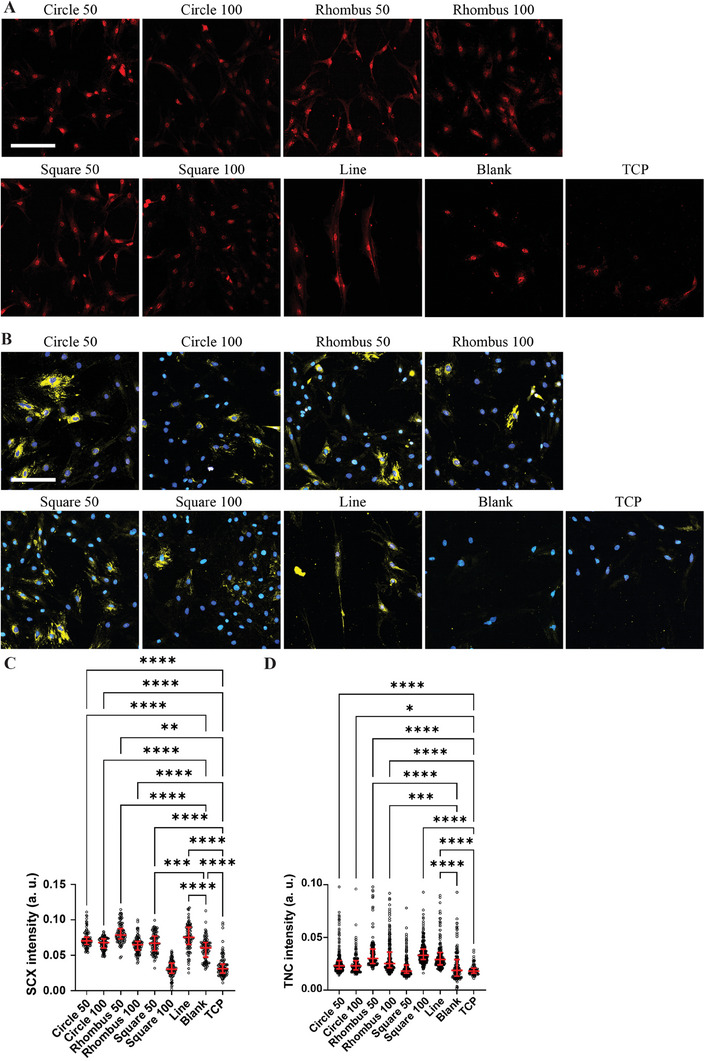
Tenogenic marker expression of tenocytes cultured on collagen fibers for 3 days. A,B) Representative immunocytochemistry images of rat tenocytes cultured on fibrous collagen micropatterns formed in microfluidic devices with different micropillar arrays and without (Blank) and on TCP. Tenocytes were stained for SCX (red; A) and TNC (yellow; B), and with DAPI to visualize cell nuclei (blue, B). Scale bars represent 50 µm and apply to all images. C,D) Quantitative analysis of nuclear SCX intensity (C) and TNC expression (D). Median values are represented by horizontal lines and interquartile ranges by error bars. *p < 0.05, **p < 0.01, ***p < 0.001, ****p < 0.0001. *N* = 3.

Next, we assessed whether the collagen micropatterns have an influence on tenogenic phenotype by analyzing the expression of the transcription factor SCX.^[^ ^27^ ^]^ Nuclear SCX expression in primary tenocytes was observed after both 1 and 3 days of culture. On day 1, levels of SCX intensity (see Section 5, Image Analysis) were significantly higher in tenocytes cultured on micropatterns of aligned collagen obtained with Line devices (fiber orientation: 50%) relative to cells cultured on collagen from Blank devices (orientation: 22%) and TCP (Figure S5B,D, Supporting Information). On day 3, we observed increased SCX levels in tenocytes cultured on micropatterns of aligned collagen obtained with Circle 50 (orientation: 60%), Rhombus 50 (orientation: 50%), Square 50 (orientation: 55%), and Line devices (orientation: 50%) relative to tenocytes cultured on other collagen micropatterns, Blank collagen substrates and TCP (**Figure** **4**A,C). These data indicate that the presence of collagen, in particular with distinct organization, supports the expression of SCX in tenocytes, especially during a longer culture period (Figure S8, Supporting Information).

Appropriate tendon ECM composition, including tendon matrix proteins such as COL‐I, COL‐III, and TNC, is imperative for maintaining tenocyte function.^[^ ^28^ ^]^ Therefore, as a functional output, we investigated the effect of collagen substrate organization on the production and deposition of the matrix glycoprotein TNC after 5 days of tenocyte culture (Figure 4B,D). Quantification of TNC mean intensity (see Section 5, Image Analysis) revealed, with the exception of the Square 50 micropattern, significantly increased deposition by tenocytes cultured on micropatterned collagen fibers compared to those cultured on the Blank substrate or the TCP control.

### 2.5 Gene Expression Profile of Tenocytes Cultured on Aligned Collagen Fibers Obtained with Circle 50 and Rhombus 50 Devices

**Figure 5:**
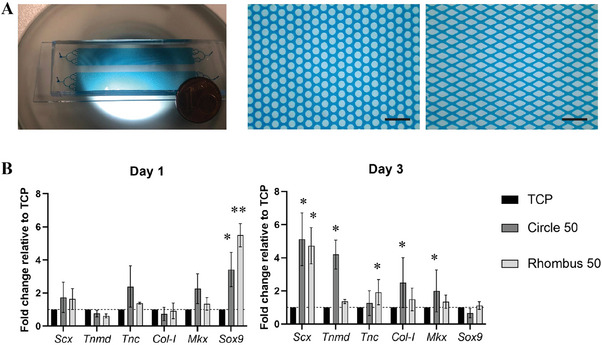
Gene expression profile of tenocytes cultured on aligned collagen fibers obtained with Circle 50 and Rhombus 50 devices with increased base area. A) Brightfield microscopy images of the total view of a microfluidic device for micropatterning a larger area (left) and zoomed‐in views showing the two micropillar arrays (middle and right). For visualization purposes, the microfluidic devices are filled with a blue liquid. Scale bars represent 200 µm. B) Bar graphs showing the expression of tenogenic genes in tenocytes cultured on Circle 50, Rhombus 50 and TCP control for 1 day (left) and 3 days (right). Values are given as fold changes relative to the expression in tenocytes cultured on TCP. Bars and error bars represent mean values and standard deviations, respectively. *p < 0.05, **p < 0.01. *N* = 3.

The results discussed so far indicate that the strongest effect on tenocyte morphology and the highest levels of nuclear SCX intensity levels were found in tenocytes cultured on Circle 50 and Rhombus 50 micropatterns. To investigate whether these collagen micropatterns also affected the maturity of tenocytes, we investigated the expression of the tenogenic genes *Scx*, Mohawk homeobox (*Mkx*), *Col‐I*, *Tnmd*, *Tnc*, and SRY‐box transcription factor 9 (*Sox9*) after 1 and 3 days of culture. For this purpose, we scaled up the corresponding two microfluidic devices for micropatterning a larger base area of now 10 mm × 45 mm, while keeping the original micropillar (hexagonal) arrangement and spacing (**Figure** **5**A), as also the height of the flow chambers. This increased area allowed the culture of a greater number of cells to obtain sufficient mRNA for real‐time quantitative PCR (RT‐qPCR) analysis.

Collagen micropatterns obtained on the increased area were comparable to micropatterns obtained using the original device design, showing a percentage of fibers oriented around 0° of 43% for the Circle 50 and 42% for the Rhombus 50 micropattern (Figure S9, Supporting Information). Gene expression results (Figure 5B) confirmed that *Scx* levels were upregulated on collagen micropatterns obtained using Circle 50 and Rhombus 50 devices relative to the TCP control as early as at day 1 and reaching statistical significance after 3 days. A similar trend was observed for the transcription factor *Mkx*, a key regulator of tendon homeostasis,^[^ ^29^ ^]^ in tenocytes cultured on Circle 50 micropatterns. Interestingly, only a mild *Mkx* upregulation, not significantly different from the TCP control, was observed from tenocytes cultured on micropatterns resulting from Rhombus 50 devices. The expression levels of *Col‐I*, a downstream target of *Mkx*,^[^ ^30^ ^]^ increased at day 3, significantly for tenocytes cultured on Circle 50, compared to those cultured on the TCP control. For *Tnmd*, a marker for later‐stage tendon differentiation,^[^ ^31^ ^]^ on day 1, the expression levels on collagen micropatterns were comparable to those on the TCP control. On day 3, significant upregulation was observed in cells cultured on Circle 50 micropatterns. *Tnc* showed a statistically significant increase when cells where cultured on micropatterns fabricated using the Circle 50 devices at day 3 relative to the TCP control. Finally, the expression levels of the chondrogenic transcription factor *Sox9*, also expressed at the tendon‐bone junction,^[^ ^32^ ^]^ were significantly higher in tenocytes cultured on Circle 50 and Rhombus 50 collagen micropatterns after 1 day but decreased to levels comparable to the control by day 3. These RT‐qPCR data indicate that tenocytes retained their mature phenotype on micropatterned collagen in contrast to traditional TCP.

### 2.6 Induction of Tenogenic Differentiation in hMSCs Cultured on Aligned Collagen Fibers Obtained with Circle 50 Devices

Because of the limited availability of primary tenocytes from humans, for the most part of the study, we used primary rat tenocytes. However, to explore the translational potential of our platform, we introduced human MSCs (hMSCs) and investigated their tenogenic differentiation potential when cultured on the aligned collagen patterns. Additionally, we explored the impact of the transforming growth factor (TGF)‐β2, alone or in combination with the collagen patterns. The members of the TGF‐β family in general and particularly TGF‐β2 are known for their role in tenogenesis.^[^ ^33^ ^]^ Cell differentiation was evaluated over an extended culture period of 14 days. On the other hand, since changes in cell phenotype tend to occur early,^[^ ^34^ ^]^ we employed a shorter culture period of 3 days when working with the rat tenocytes.

**Figure 6:**
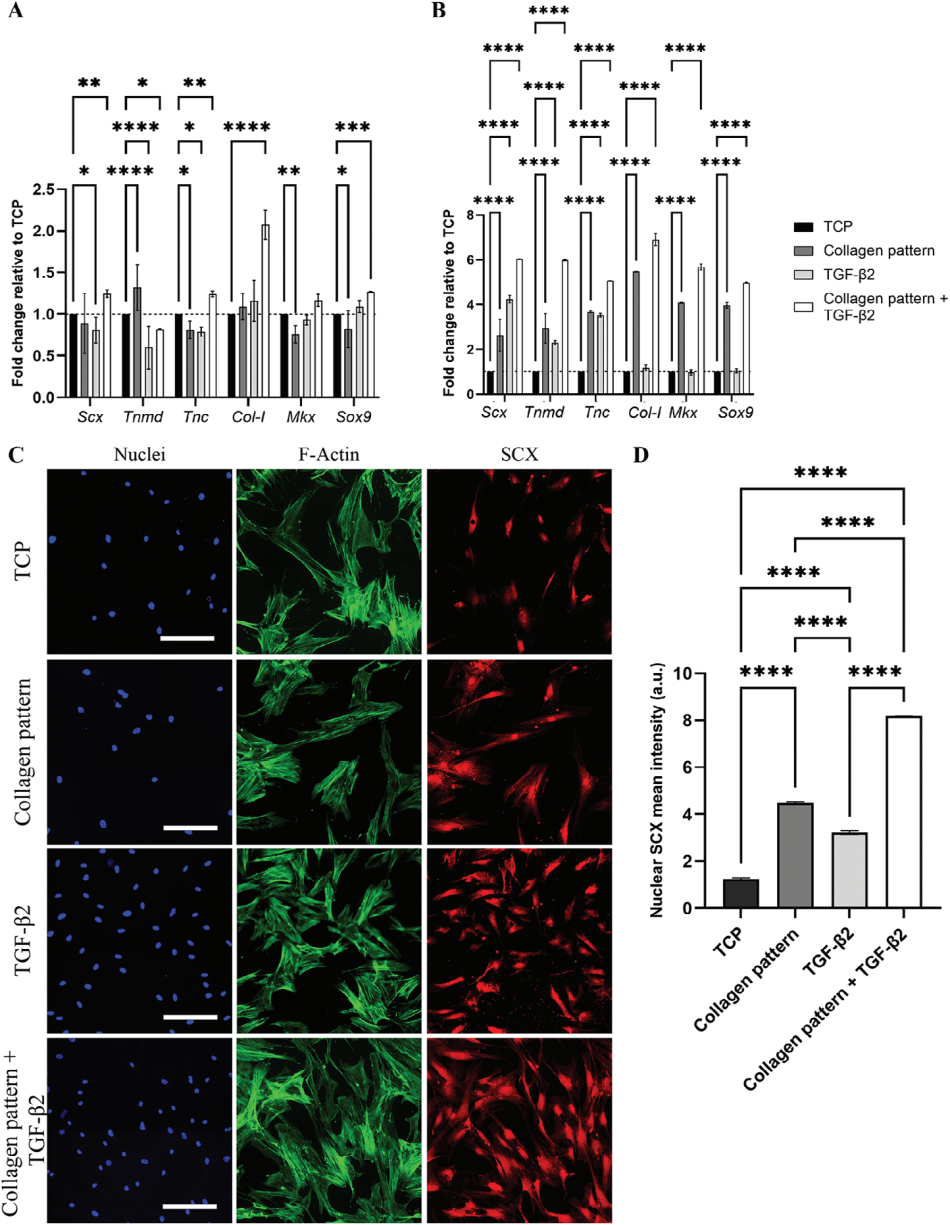
Tenogenic differentiation of hMSCs cultured on aligned collagen fibers obtained with Circle 50 devices. A,B) Bar graphs showing the expression of tenogenic genes in hMSCs cultured on TCP, on aligned collagen patterns obtained with Circle 50 devices, on TCP combined with 20 ng mL^−1^ TGF‐β2 and on collagen patterns in combination with the same concentration of TGF‐β2 for 3 days (A) and 14 days (B). Values are given as fold changes relative to the expression in hMSCs cultured on TCP. Bars and error bars represent mean values and standard deviations, respectively. *p < 0.05, **p < 0.01, ***p < 0.01, ****p < 0.0001. *N* = 3. C) Representative immunocytochemistry images of hMSCs cultured on TCP, aligned collagen patterns, TCP supplemented with 20 ng mL^−1^ TGF‐β2 and on collagen pattern in combination with TGF‐β2 for 14 days. hMSCs were stained with DAPI to visualize cell nuclei (blue) and phalloidin to visualize F‐actin (green), and for SCX (red). Scale bars represent 50 µm and apply to all images. D) Quantitative analysis of nuclear SCX intensity. Bars and error bars represent mean values and standard deviations, respectively. ****p < 0.0001. *N* = 3.

First, we investigated the impact of aligned collagen patterns obtained with the Circle 50 device on the expression of tenocyte‐related genes in the hMSCs cultured on them. For this, we cultured the cells on the aligned collagen patterns and on TCP, in both cases without and with the supplementation of 20 ng mL^−1^ TGF‐β2. After 3 days of culture, we observed an upregulation of *Scx*, *Tnc*, *Col‐I*, *Mkx*, and *Sox‐9* expression levels in cells cultured on the collagen patterns combined with TGF‐β2 compared to the other conditions (**Figure** **6**A). In case of *Col‐I*, this upregulation was around twofold. Relative to TCP, *Tnmd* was significantly upregulated in cells cultured on collagen patterns without the addition of TGF‐β2 to the culture medium, whereas its expression was downregulated in the other conditions. After 14 days of culture, compared to only TCP, *Scx* expression was upregulated in the other conditions (Figure 6B); cells cultured only on the aligned collagen patterns showed a more than twofold‐*Scx* upregulation, on TCP in combination with TGF‐β2 a roughly fourfold up‐regulation, and on collagen combined with TGF‐β2 an around sixfold increase in *Scx* expression. Similarly, *Tnmd* expression levels exhibited a more than twofold increase in cells cultured on TCP together with TGF‐β2 and on collagen patterns, and an around sixfold increase in the combined culture condition. Compared to TCP, *Tnc* expression levels were more or less than fourfold upregulated in the other conditions. *Col‐I* showed a near‐to‐six‐/sevenfold up‐regulation in cells cultured in both collagen pattern conditions, and comparable expression levels for both TCP conditions. The expression patterns of *Mkx* and *Sox‐9* mirrored those of *Col‐I*.

To assess protein expression, we evaluated the nuclear expression of SCX (Figure 6C). The quantification of SCX nuclear signals revealed that there is, compared to only TCP, a significant increase in the other conditions (Figure 6D). In addition, hMSCs cultured on the aligned collagen pattern without and with the supplementation of TGF‐β2 exhibited a higher SCX expression compared to TCP cultures supplemented with TGF‐β2. The combination of the collagen pattern and TGF‐β2 demonstrated a significant increase in SCX expression compared to the other conditions.

Taken together, these results highlight the combined impact of the aligned collagen pattern and TGF‐β2 on enhancing the expression of key tenogenic markers and SCX expression. This suggests the potential of this combination in promoting tenogenic differentiation.

### 2.7 Establishment of 3D Cultures in Circle 50 Device

Compared to solutions where the tendon cells are completely embedded in a (collagen) hydrogel, the cell‐instructive micro‐/nanotopographical culture interface presented in this study allows an easier workflow concerning cell seeding, observation, and harvesting for further downstream processing. However, 3D models provide a physiologically more relevant environment for studying cell–cell and cell–ECM interactions than their 2D counterparts. Therefore, we expanded our study by a short feasibility check concerning a volumetric model based on the same piggyback approach as already taken for the biointerface model mostly investigated in this study. For this, we perfused the microfluidic device with the stabilized collagen‐I solution, which now already contained the rat tenocytes. Then, the 3D model was cultured for a period of 3 days. Confocal fluorescent microscopy imaging revealed that the rat tenocytes were truly embedded within the collagen matrix and exhibited alignment along the collagen fibers (Figure S10, Supporting Information). This observation supports the idea that such a 3D model could mimic the native 3D tissue microenvironment.

### 2.8 Correlation between Cell Morphology Descriptors, SCX/Scx Levels, and Genetic Profile Induced by Collagen Micropatterns

**Figure 7:**
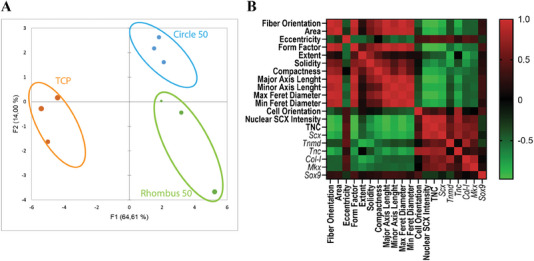
PCA of cell shape descriptors, and protein and gene profiles. A) PCA plot showing the clustering of samples analyzed, based on similarity according to the first principal component (representing 64.61% of total variance) and the second principal component (representing 14.0% of total variance). Each dot represents a biological replicate. Colored ellipses have been manually added to the plot to visualize the clusters. B) Heat map showing the correlation between cell descriptors analyzed (for the exact definition and explanation of the cell morphology descriptors listed, see the documentation of the Measurement module in the CellProfiler manual under https://cellprofiler‐manual.s3.amazonaws.com/CellProfiler‐4.2.4/modules/measurement.html).

Based on our preceding observations that collagen micropatterns affected tenocyte morphology, protein, and gene levels, these parameters were subjected to a principal component analysis (PCA) to identify potential correlations between them (**Figure** **7**). The analysis resulted in 20 principal components. The PCA plot, based on the first principal component covering 64.6% of the total variability and the second one covering 14.0%, shows the formation of three main clusters, corresponding to the two different micropatterns, which are Circle 50 and Rhombus 50, and the TCP control, analyzed according to the quantified cell descriptors (Figure 7A). The correlation matrix shows that nuclear SCX intensity had a strong correlation with cell morphology parameters—cell area, form factor, compactness (i.e., the mean squared distance of an object's/a cell image's pixels from the centroid/geometric center of the object/cell divided by its area), major axis length, maximal Feret diameter, and minimal Feret diameter (i.e., the maximum and minimum distance between two parallel lines tangent on either side of the outline of an object/cell) and minor axis length—as well as with the orientation of collagen fibers and *Scx* and *Tnc* gene expression (Figure 7B). We observed a positive correlation between *Scx* and SCX and between *Tnc* and TNC expression, while a negative correlation was observed between *Scx* expression and cell shape descriptors, such as form factor, axis length, and Ferret diameter. A positive correlation was observed between *Col‐I* and *Tnmd* gene expression, while a negative correlation exists between the expression of these two genes and cell solidity/“protrusivity” (i.e., the object/cell area dived by the convex hull area of the object/cell), eccentricity, and *Sox9* expression. The PCA data showed that tenocytes behaved differently with respect to cell morphology and tenogenic marker expression when they are cultured on collagen micropatterns compared to TCP.

### 2.9 Focal Adhesion Length in Tenocytes Culture on Aligned Collagen Fibers Obtained with Circle 50 and Rhombus 50 Devices

**Figure 8:**
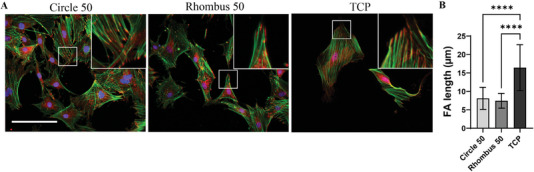
FAs of tenocytes cultured for 3 days on fibrillar collagen micropatterns and TCP. A) Representative fluorescence microscopy images showing tenocytes stained with DAPI to visualize cell nuclei (blue) and phalloidin to visualize F‐actin (green), and for vinculin (red). Scale bar represents 50 µm and applies to all images. Insets in the upper right corners show enlarged views of the areas delineated by the white boxes. B) Quantification of FA length in tenocytes cultures on the different substrates. Bars and error bars represent mean values and standard deviations, respectively. ****p < 0.0001. Statistical analysis was performed on more than five cells of each of the three substrate groups in three independent experiments per group/*N* = 3.

The formation of focal adhesions (FAs) by cells has been shown to be dependent on the topographical features of the surface they are exposed to.^[^ ^8^, ^27^, ^35^ ^]^ Following attachment, fibroblasts tend to form stress fibers on stiff substrates,^[^ ^36^ ^]^ which require the recruitment of different FA proteins, including vinculin, to form mature contact points.^[^ ^37^ ^]^ Therefore, adhesion of tenocytes to different substrates was investigated using vinculin staining, together with cytoskeleton (F‐actin) and nucleus staining, after 1 and 3 days of culture (Figure S11, Supporting Information, and **Figure** **8**, respectively). We observed that tenocytes cultured for 3 days on micropatterned collagen fibers have significantly shorter vinculin‐containing FAs compared to tenocytes cultured on TCP (Figure 8B). This difference in FA length confirmed that the cells are responding differently to the substrates they are cultured on. The difference in FA length might be related to the available area on the substrates, which is more restricted on the micropatterned fibers^[^ ^38^ ^]^ compared to TCP.

### 2.10 Cell Phenotype, and Protein and Gene Expression Analysis of Tenocytes Cultured on Non‐Fibrillar Collagen Micropatterns and on Collagen Fiber Replicas in PS

**Figure 9:**
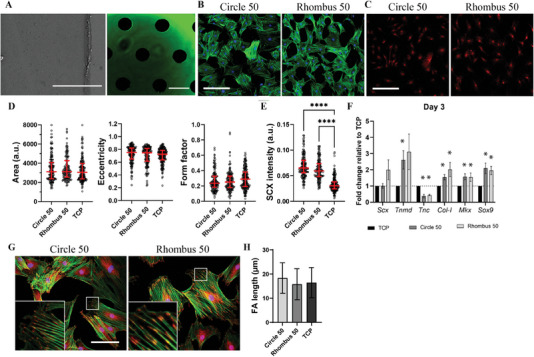
Characterization of non‐fibrillar collagen surfaces and tenocytes cultured on them for 3 days. A) SEM (left) and label‐free fluorescence microscopy images (obtained using reflection mode at 488 nm; right) of non‐fibrillar collagen surfaces obtained using Circle 100 and Circle 50 devices, respectively, but at a 100 times flower flow rate. Scale bars represent 50 and 100 µm, respectively. B,C) Representative fluorescence microscopy images of primary rat tenocytes cultured on non‐fibrillar collagen‐coated surfaces for 3 days and stained with DAPI to visualize cell nuclei (blue; B) and phalloidin to visualize F‐actin (green; B), and for SCX (red; C). Scale bars represent 50 µm and for both subfigures apply to both images. D) Quantitative analysis of cell shape parameters, including cell area (left), cell eccentricity (middle), and form factor (right). Median values are represented by horizontal lines and interquartile ranges by error bars. E) Quantitative analysis of SCX nuclear intensity. Median values are represented by horizontal lines and interquartile ranges by error bars. ****p < 0.0001. *N* = 3. F) Gene expression levels of tenocytes cultured on non‐fibrillar collagen‐coated surfaces for 3 days. Values are given as fold changes relative to the expression in tenocytes cultured on TCP. Bars and error bars represent mean values and standard deviations, respectively. *p < 0.05. *N* = 3. G) Representative fluorescence microscopy images showing tenocytes stained with DAPI to visualize cell nuclei (blue) and phalloidin to visualize F‐actin (green), and for vinculin (red). Scale bar represents 50 µm and applies to both images. Insets in the lower left corners show enlarged views of the areas delineated by the white boxes. H) Quantification of FA length in tenocytes cultured on the different non‐fibrillar collagen substrates and TCP. Bars and error bars represent mean values and standard deviations, respectively. Statistical analysis was performed on more than five cells of each of the three substrate groups in three independent experiments per group/*N* = 3.

**Figure 10:**
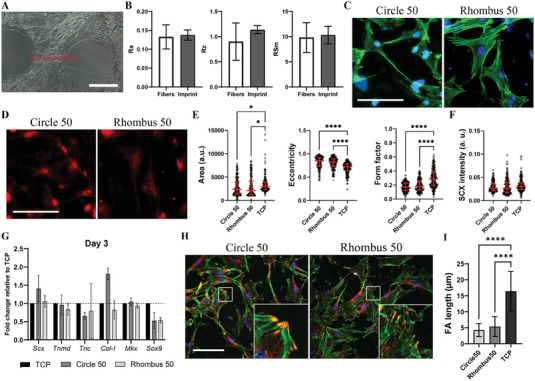
Characterization of collagen fiber replicas in PS and tenocytes cultured on them for 3 days. A) Representative confocal laser profilometry image of the fiber replica surface. Red dotted line indicates the path chosen for the characterization of height profile. Scale bar represents 50 µm. B) Quantification of arithmetic roughness average (Ra; left), maximum roughness (Rz; middle) and mean width of profile elements of the collagen fibers and their respective replicas in PS (RSm; right). *N* = 3. C,D) Representative fluorescence microscopy images of primary rat tenocytes cultured on the replicas for 3 days. Tenocytes were stained with DAPI to visualize cell nuclei (blue; C) and phalloidin to visualize F‐actin (green; C), and for SCX (red; D). Scale bars represents 50 µm and for both subfigures apply to both images. E) Quantitative analysis of cell shape parameters, including cell area (left), cell eccentricity (middle), and form factor (right). Parameters of tenocytes cultured on TCP are shown as a control. Median values are represented by horizontal lines and interquartile ranges by error bars. *p < 0.05, ****p < 0.0001. *N* = 3. F) Quantification of SCX nuclear intensity levels. Median values are represented by horizontal lines and interquartile ranges by error bars. *N* = 3. G) Gene expression levels of tenocytes cultured on collagen fiber replicas in PS after 3 days. Values are given as fold changes relative to the expression in tenocytes cultured on TCP. Bars and error bars represent mean values and standard deviations, respectively. *N* = 3. H) Representative fluorescence microscopy images showing tenocytes stained with DAPI for cell nuclei (blue) and phalloidin for F‐actin (green), and for vinculin (red). Scale bar represents 50 µm and applies to both images. Insets in the lower right corners of the images show enlarged views of the areas delineated by the white boxes. I) Quantification of the FA length in tenocytes cultured on the different PS replica substrates and TCP. Bars and error bars represent mean values and standard deviations, respectively. ****p < 0.0001. Statistical analysis was performed on more than five cells of each of the three substrate groups in three independent experiments per group/*N* = 3.

The results obtained so far showed a distinct effect of fibrous collagen micropatterns on tenogenic morphological characteristics and gene expression. We then set out to further investigate whether this effect is derived from the biochemical nature of collagen or its structural characteristics, that is, fibrillar organization. For this, we pursued two approaches (**Figures** **9** and **10**). First, we prepared non‐fibrillar collagen substrates using the Circle 50 and Rhombus 50 devices but at a 100 times lower flow rate than for fibrillar collagen. SEM and fluorescence microscopy images showed a flat, collagen‐coated surface, without fiber formation (Figure 9A). Second, we used imprinting by means of a mold in the form of an elastomeric intermediate replica of the fibrous collagen micropatterns to transfer their topography into a PS film (Figure S12A, Supporting Information). Confocal laser profilometry images suggested that the collagen microstructures were replicated with high fidelity (Figure 10A). Quantification of the topographical profile showed comparable values in terms of surface roughness for the original microstructures and their PS replicas, confirming that the collagen fibers were reproduced with fidelity (Figure 10B).

Rat tenocytes were cultured for 1 and 3 days, after which their morphology was analyzed. Tenocytes cultured on non‐fibrillar collagen‐coated surfaces micropatterned with Circle 50 and Rhombus 50 devices for 3 days exhibited a spread morphology (Figure 9B). Values for cell area, eccentricity and form factor at day 3 were comparable to those obtained when cells were cultured on the TCP control (Figure 9D). This demonstrates that in the absence of a fibrillar structure collagen‐coated surfaces do not affect/change cell morphology in this comparison. In contrast, tenocytes cultured on collagen fiber replicas in PS for 3 days showed a significant decrease of cell area and circularity and a significant increase of cell elongation for both Circle 50 and Rhombus 50 micropatterns (Figure 10C,E). These results indicate that fibrillar structure and organization, rather than collagen biochemistry, affect the morphological response and cytoskeletal reorganization of tenocytes.

Next, we determined the expression of tenocyte markers after culture on these different substrates. Quantification of SCX expression showed significantly higher nuclear levels in tenocytes cultured for 3 days on non‐fibrillar collagen‐coated surfaces than on the TCP control (Figure 9C,E). When the cells were cultured on PS replicas of fibrillar collagen, nuclear SCX levels were comparable to those of tenocytes cultured on TCP (Figure 10D,F). These data show that, unlike tenocyte morphology, the SCX expression is influenced by the presence of collagen biochemistry on the flat surface rather than the collagen's fibrillar structure in PS. RT‐qPCR analyses of rat tenocytes showed a similar result with some exceptions. For tenocytes cultured on non‐fibrillar collagen‐coated surfaces, *Scx, Tnmd*, *Col‐I, Mkx*, and *Sox9* were upregulated after both 1 and 3 days relative to the TCP control (except in one case where the expression was the same) (Figure S13, Supporting Information, and Figure 9F, respectively), with *Tnmd* (for Circle 50 micropatterns), *Col‐I*, *Mkx*, and *Sox9* expression significantly increased at day 3. *Col‐I* was also significantly increased at day 1 in tenocytes cultured on the collagen‐coated Rhombus 50 surfaces. Significant upregulation was observed for *Mkx* on day 1, followed by a slight decrease at 3 days. *Tnc* expression significantly decreased on both surfaces relative to the control at day 3. By comparison, tenocytes cultured for 1 day on collagen fiber replicas in PS showed expression levels of *Scx*, *Tnmd*, *Tnc*, *Col‐I*, *Mkx*, and *Sox9* similar to those observed on TCP (Figure S12B, Supporting Information). Moderate upregulation was observed for *Scx* and *Col‐I* genes on PS replicas of Circle 50 collagen micropatterns after 3 days of culture, while *Sox9* gene expression was downregulated on replicas of Circle 50 and Rhombus 50 micropatterns (Figure 10G), similar to what we observed on collagen micropatterns (Figure 5B). No statistical differences were found for the different genes analyzed compared to TCP.

We then quantified the FA length of tenocytes cultured for 3 days on non‐fibrillar collagen or PS replicas in relation to the TCP control (for FA formation in tenocytes cultured on the same substrate types but for 1 day, see Figure S14, Supporting Information). No significant differences were found comparing non‐fibrillar collagen to TCP (Figure 8G,H), while significantly shorter vinculin‐containing FAs were measured in tenocytes cultured on PS replicas (Figure 9H,I). As already indicated in Section 2.7, this difference in FA length might be related to the available area on the substrates,^[^ ^38^ ^]^ which is more restricted in case of the collagen fibers and their replicas compared to TCP and the non‐fibrillar collagen.

PCA was performed to determine potential correlations based on the same cell descriptors as already in Section 2.6 (Figure S15, Supporting Information). The PCA plot, based on the first and second principal components together covered 74.28% of the total variance of the dataset. This analysis showed the formation of four main clusters, indeed confirming a different behavior when tenocytes were cultured on non‐fibrillar collagen compared to replicas of collagen fibers in PS.

## 3 Discussion

An important challenge is to recreate the ECM environment of the native tissue in order to regulate cell behavior and function. In this context, producing aligned collagen fibers that mimic both the biochemical nature and the microstructural properties of tendon ECM is a promising approach for supporting the maintenance of tenocyte phenotype and facilitating tendon repair and regeneration.^[^ ^39^ ^]^ Here, we developed a microfluidic piggyback platform that enables the control of the micropatterned formation and alignment of collagen fibers, for example, on the bottom of culture dishes. This control was achieved by using arrays of micropillars with varying shape and interpillar spacing. Through the resulting microchannel networks between the micropillars a collagen solution could be flowed. In the already mentioned study by Lee et al., aligned collagen was obtained in microfluidic channels with widths of ≤100 µm, whereby the degree of collagen alignment was lower in microchannels of larger size.^[^ ^21^ ^]^ In our microfluidic platform, we also observed a clear orientation of collagen fibers in the direction of the long axis of the microchannels. For one and the same volumetric flow rate, alignment was higher for the smaller spacing between the micropillars, which means narrower microchannels between them, consequently leading to higher average flow speeds (Figure 1B). Minimum 50 µm wide microchannels resulted in orientations >50%, minimum 100 µm wide microchannels in orientations <38% (Figure 2). As for the various channel network designs the fiber orientation followed the channel and flow direction, there are good reasons to believe that this should be also possible for other channel patterns, such as arch‐type ones, representing other tissue architectures. Concerning culture area‐related throughput, one can get an idea about what could be possible when looking on the scaled‐up 10 mm × 10 mm areas of patterned aligned collagen from the gene expression experiments presented in Section 2.5. This asks for a follow‐up study, similar to questions regarding the resolution of the patterning method in combination with finer channel networks. Apart from the effect of flow on collagen alignment, what might be interesting to further investigate is the effect of the (near) sidewall of the microfluidic channels on the formation and alignment of the collagen fibrils and fibers in terms of steric hindrance of and electrostatic interactions with the collagen molecules and their assemblies.

Apart from electrospinning based approaches to create anisotropic and aligned PLLA‐collagen, PLLA, or polycaprolactone (PCL) fiber matrices as culture substrates or scaffolds for fibroblasts, tendon stem/progenitor cells, and tendon fibroblasts, respectively,^[^ ^18^, ^29^, ^35^ ^]^ other techniques have been applied for the creation of such matrices. For example, Lehner et al. reported on rat tendon‐derived cells mixed with collagen type I solution that was then spread between two silk sutures pinned with insect pins in rows on PDMS‐coated Petri dishes.^[^ ^40^ ^]^ Xu et al. reported that in a mixture of collagen I, iron oxide nanoparticles (IOPs) and human tendon stem/progenitor cells the IOPs were aligned under a remote magnetic field, and that following the gelation of the collagen a stable and anisotropic nanocomposite hydrogel was formed.^[^ ^41^ ^]^

Collagen fiber alignment affects cell morphology, as demonstrated in other studies,^[^ ^6^, ^28^, ^42^ ^]^ although its effect on the maintenance of tenogenic phenotype was not assessed yet. For example, Yin et al. showed that tendon progenitor cells cultured on aligned electrospun PLLA nanofibers displayed an elongated morphology.^[^ ^29^ ^]^ In line with these findings, in our study, we observed that culturing tenocytes for 3 days on micropatterns of aligned collagen resulted in a more elongated cell morphology (i.e., smaller area, higher eccentricity, and lower form factor). This effect was dependent on the extent of collagen fiber alignment, with cells cultured on micropatterns derived from narrower microchannels, as mentioned in the results section, exhibiting a morphology of early‐passage primary tenocytes.^[^ ^4^, ^9^ ^]^ We also observed that after the initial adhesion on aligned collagen fibers (day 1), cells undergo a change in cell shape from a more spread to a smaller and more elongated morphology after 3 days of culture, confirming that the cytoskeleton adapts to the topographical environment.^[^ ^9^, ^30^, ^31^ ^]^

Cell morphology directly links with cell fate and differentiation.^[^ ^32^ ^]^ Accordingly, we showed that more elongated cells directly correlate with higher nuclear SCX levels and increased deposition of the ECM marker TNC (Figure 4). Conversely, rounder cells (eccentricity and form factor closer to 0 and 1, respectively), observed on TCP and rather randomly oriented collagen obtained from the Blank devices, correlated with a reduced SCX expression and decreased TNC production. These results suggest that the interplay between the microstructure of the aligned collagen and actin cytoskeletal network can actively modulate cell fate. Whether and how the Rho/ROCK signaling pathway^[^ ^6^, ^31^, ^32^, ^43^ ^]^ plays a role here will be interesting to investigate in future studies.

The expression of tendon‐related genes confirmed the tenogenic phenotype upon culture on micropatterns of aligned collagen (Figure 5). After 3 days of culture, compared to the TCP control, the marker genes *Scx*, *Tnmd*, *Col‐1*, and *Mkx* were upregulated in cells cultured on aligned collagen from the Circle 50 device (orientation: 60%), and *Scx* and *Tnc* in cells cultured on aligned collagen from the Rhombus 50 device (orientation: 50%). Similarly, culturing tenocytes on arrayed synthetic microtopographies was shown to positively modulate the expression of different tenogenic markers, including *Scx*, *Mkx*, *Tnmd*, and *Col‐I*.^[^ ^9^ ^]^ In the corresponding study, Vermeulen et al. reported an up to fourfold increased expression of *Tnmd* and mild but significantly higher *Mkx* and *Col‐I* levels compared to TCP. In line with this, in the same study, also aligned grooves in the micrometer range were shown to increase the expression of both *Tnmd* and *Col‐I*, similarly as observed in this study for aligned collagen fibers. An increased expression was also observed for the chondrogenic marker *Sox9*, which, as previously reported, could be dependent on the time point analyzed,^[^ ^4^ ^]^ here at an early stage of culture (day 1). Interestingly, levels of *Sox9* decreased roughly to control level by day 3. This might be related to the increased *Scx* levels we measured at the later time point, since a previous study suggested that *Scx* expression can repress *Sox9* expression and the corresponding transcriptional cascade.^[^ ^27^ ^]^ Our findings are consistent with further previous studies. Tendon progenitor cells showed higher expression of tenogenic markers on aligned PLLA nanofibers than on randomly oriented fibers.^[^ ^29^ ^]^ Similarly, aligned electrospun PCL fibers induced higher expression not only of tenocyte‐related markers, such as *Mkx* or *Col‐1* and ‐*3*, but also of ECM markers, such as biglycan and *Dcn*, in early‐passage tendon fibroblasts when compared to randomly oriented fibers.^[^ ^35^ ^]^

The culture durations chosen for the primary rat tenocytes in this study and being similar to that stated in other papers in the field^[^ ^12^, ^44^ ^]^ allow to evaluate the initial response of the cells to the aligned collagen patterns and the ability of the collagen patterns to maintain the cells’ tenogenic phenotype within the chosen time periods. By including hMSCs and implementing long(er)‐term cultures, we could investigate the induced cell differentiation potential resulting from the collagen patterns. This dual approach provides a more comprehensive understanding of the effect of the collagen patterns on cell behavior, which could, among others, facilitate the development of more effective TERM strategies in the medium and long term. To get an even more conclusive overview of the capacities of the proposed microfluidic platform and the aligned collagen patterns it can create, future efforts in this direction should include the confirmation of phenotypical maintenance in long(er)‐term cultures of the rat tenocytes from this study, and also first cultures with human primary tenocytes. Another important aspect to be investigated in this context is the potential loss of the cell morphology (i.e., the change from a spindle‐ to a more stellate‐like cell shape) and cellular phenotype in consecutive passages as observed on flat TCP surfaces in past studies.^[^ ^4^, ^9^ ^]^ By three‐dimensionally embedding the rat tenocytes within the collagen, we made a proposition for the development of a miniaturized 3D tendon in vitro model.

Clustering analysis indicated a strong negative correlation between tenocyte protein and gene expression and a number of cell morphology descriptors. This observation implies a link between tenocyte fate and morphology. Indeed, cell shape is known to influence several molecular pathways through cytoarchitectural rearrangement, representing a relevant factor controlling cell fate and commitment. Moreover, tenocyte shape and protein and gene expression are different depending on whether the cells are cultured on TCP or on aligned collagen fibers obtained with Circle 50 and Rhombus 50 platforms (Figure 7A), reflecting a different cell phenotype according to the substrate the cells are cultured on. To investigate whether these results were due to the biochemistry of (molecular) collagen, the microstructure of its fibrils/fibers or both, we cultured tenocytes on non‐fibrillar collagen or PS replicas of aligned collagen fibers to decouple the chemical from the structural characteristics. The actual fiber structure, in the absence of collagen, controlled tenocyte shape. However, the presence of collagen, even in its non‐fibrillar form, positively affected SCX nuclear expression.

When analyzing the tenogenic gene expression, we observed that tenocytes cultured on non‐fibrillar collagen‐coated surfaces for 3 days displayed an increased expression of *Tnmd*, a regulator of tenocyte proliferation that promotes the maintenance of the differentiated phenotype.^[^ ^45^ ^]^ In MSCs cultured on collagen‐coated surfaces, the upregulation of *Tnmd* was associated with the active *Wnt/β‐catenin* signaling pathway, which is known to play a role in the expression of *Scx* and *Mkx*.^[^ ^4^ ^]^ In our study, unlike *Tnmd*, a moderate but not significant upregulation of *Scx* expression was observed when tenocytes were cultured on non‐fibrillar collagen relative to the TCP control. This difference may be related to the TGF‐β signaling activity as in vitro studies demonstrated that this pathway affects the expression of tenogenic markers, namely *Scx* and *Mkx*, and also of the tendon matrix proteins COL‐I and TNC.^[^ ^37^ ^]^ This suggests the sensitivity of these markers to cytoskeletal reorganization in tenocytes, as a result of the microstructural properties of the substrate on which they are cultured.^[^ ^31^ ^]^ These results confirm the role for both the biochemical nature and fibrillar structure in supporting the tenogenic phenotype and are in line with previous work by our group in the context of the osteogenic phenotype.^[^ ^46^ ^]^

When quantifying the length of vinculin‐containing FAs in tenocytes, longer FAs were observed in cells cultured on non‐fibrillar collagen‐coated surfaces and TCP compared to cells cultured on aligned collagen fibers and their replicas in PS. In line with these observations, it has been previously reported that downregulation of SCX expression in tenocytes results in longer vinculin‐containing FAs,^[^ ^47^ ^]^ which is different from our observation though (Figures 9F,H and 10G,I). Further research is required regarding the mechanotransduction mechanisms underlying the biological responses of tenocytes to different substrates.

Taken together, the platform developed here represents a simple and flexible method for producing collagen fibers with different extents of alignment according to the shape of and spacing between arrayed micropillars included in the flow chamber of a microfluidic device temporarily mounted on a culture substrate and flown through by a collagen solution. This platform offers a useful tool to easily investigate the interactions between tenocytes and the microfluidically produced collagen matrix, which may serve as an inspiration for developing novel strategies for repair and regeneration of damaged tendon tissue. Further research may combine the inclusion of other biochemical factors, such as glycosaminoglycans, into aligned collagen fibers, as these factors are known to be present in tendon ECM.^[^ ^48^ ^]^ Alternatively, the platform could be used to more reliably reconstitute the diversity of collagenous ECM in different cases, such as differences between the tendon‐to‐bone and tendon‐to‐muscle interface and differences between a healthy and a diseased tendon.^[^ ^35^ ^]^

Overall, this study demonstrates that micropatterns of aligned collagen fibers provide an instructive microenvironment for tenocytes. Culturing tenocytes on aligned collagen fibers may prevent the loss of phenotypic tenocyte markers usually observed within 5 passages, which might allow improved clinical outcomes in TE applications. The presented platform and the resulting knowledge can help the rational design of materials‐driven strategies for tendon regeneration.

## 4 Conclusion and Outlook

In conclusion, we developed a microfluidic platform to fabricate collagen fiber micropatterns with different degrees of fiber alignment. These micropatterns were used to study the interaction between the collagenous matrix and primary rat tenocytes as well as hMSCs. We demonstrated that the retention of a more elongated tenocyte morphology and the maintenance of the tenogenic phenotype were strongly supported by the fibrous collagen patterns. Further developments of similar platforms might, in not too distant future, be able to delay tenocyte drift or at some point even reverse it.

## 5 Experimental Section

### Design, Fabrication, and Characterization of the Microfluidic Platform

The microfluidic devices of the platform with the flow chamber‐integrated micropillar arrays were designed using the AutoCAD computer‐aided design software (AutoDesk). Designs included micropillars with different shapes—circle, rhombus, square, and line—and spacing between them—50 and 100 µm. The features were printed on a film‐based photomask. The mask was used to fabricate 35 µm high SU‐8 molds on silicon wafers by UV photolithography. The molds featured the inverse/“negative” topography of the micropillars and the microfluidic channel networks in between. Dimensional characterization of the molds’ geometrical features was performed using a VK‐X250 3D confocal laser scanning microscope‐based optical profilometer (KEYENCE).

The molds were then used to fabricate microfluidic devices by PDMS (Sylgard 184, Dow) casting. The PDMS mixture with a base elastomer‐to‐curing agent ratio of 10:1 was poured onto the molds, degassed in a vacuum chamber and cured at 80 °C for 1 h in an oven. The cured slabs from PDMS containing multiple flow chambers with associated inlet and outlet channels were then detached from the molds, the top parts of the microfluidic devices were cut out of the PDMS slabs with a razor blade and fluidic connection ports were punched in the cut‐out PDMS parts using a 730‐µm‐diameter puncher (SYNEO). The freshly cured PDMS parts and microscopy glass slides were cleaned in 70% ethanol and then allowed to reversibly/peelably bond to each other at 80 °C overnight in an oven.

### CFD Simulation

CFD simulations of the flow fields in the micropillared flow chambers were performed using the COMSOL Multiphysics finite element modeling software (COMSOL; version 5.4). Because of the extruded/“2.5D” geometry of the flow chambers, the CFD simulations were set up as simplified, 2D simulations. The simulations were run/calculated as laminar and steady/stationary flow problems according to the Navier–Stokes equation(s). The boundary conditions at the chamber sidewalls and micropillar walls were set to “no slip.” The collagen solution flown through the chambers was approximated as a (incompressible) Newtonian fluid that concerning its physical/inertial and rheological properties is identical to water at room temperature (RT). The volume flow rate at the outlet of the chamber was set to 50 µL min^−1^. The calculated flow fields were translated into velocity heat maps including flow lines.

### Microfluidic Preparation and Characterization of the Collagen Micropatterns

All subsequent steps for the preparation of the collagen micropatterns were performed under sterile conditions. Following a sterilization with 70% ethanol, the inner surfaces of the PDMS top parts were coated with 1% v/v BSA solution by flowing the solution into the reversibly bonded assemblies of the top parts with the glass slides and incubating it there at RT for 20 min. The BSA coating of the PDMS parts blocked them against collagen binding during the later collagen micropatterning. Collagen bound also to the PDMS would have resulted in the collagen patterns on the glass substrates to be (partly) removed together with the top parts in the next step. After the BSA passivation, the PDMS parts were detached from the glass slides and placed in a high(‐wall) glass‐bottom Petri dishes with a diameter of 35 mm (µ‐Dish, ibidi) where they formed perfusable/microfluidic devices upon starting a negative pressure‐driven flow. Bovine type I collagen (PureCol, Advanced BioMatrix; 3.0 mg mL^−1^; pH = 2) was mixed with one part of 10× phosphate‐buffered saline (PBS) and neutralized to physiological pH with sterile 0.1 m sodium hydroxide. The solution was diluted in sterile water to a final collagen concentration of 1.5 mg mL^−1^. In each case, the collagen solution was flown over the glass bottom of the dish by pulling it through the microchannel network between the micropillars through a negative pressure applied via the outlet of the microfluidic device. Therefore, a 1 mL syringe filled with the collagen solution and an empty 1 mL syringe were connected to the microfluidic device's inlet and outlet, respectively, through tubing with an inner diameter of 500 µm and an outer diameter of 1.5 mm (Tygon, SynVivo). Thereby, the tubing was press‐fitted into the inlet and outlet ports of the platforms. The empty syringe was mounted in a syringe pump (World Precision Instruments), which was then started to act on the syringe in a drawing‐up mode. From the time point when the flow chamber was completely filled with the collagen solution, the solution was allowed to flow through the chamber for 1 min. A flow rate of 50 µL min^−1^ was used to prepare glass bottom substrates with collagen fibers, while a flow rate of 0.5 µL min^−1^ was used for non‐fibrillar collagen substrates. After the perfusion of the chamber with the collagen solution, the collagen was allowed to polymerize in an incubator at 37 °C in a normal atmosphere overnight. Next, the PDMS part was removed from the dish‐integrated substrate, which was then ready for characterization and cell culture.

The collagen micropatterns were analyzed using a JSM‐IT200 InTouchScope scanning electron microscope (Jeol). Briefly, collagen samples were fixed in glutaraldehyde (Electron Microscopy Sciences) at 4 °C overnight, rinsed with ultrapure water and then dehydrated in a series of solutions of ethanol in distilled water with increasing ethanol concentrations (50%, 60%, 70%, 80%, 90%, and 100% v/v; 5 min per step, each step repeated twice). Finally, the samples were incubated with hexamethyldisilazane for 40 min and air‐dried. The dehydrated samples were first mounted on aluminum stubs and then coated with a thin gold layer using a Q150T ES sputter coater (Quorum). The samples were imaged at an acceleration voltage of 10.0 kV and at a working distance of 6.9 mm and 200× magnification or a working distance of 10.7 mm and 1000× magnification. Collagen fiber orientation was analyzed using the “Directionality” plugin in ImageJ (https://imagej.nih.gov/ij/), which is based on Fourier component analysis.

### Preparation and Characterization of the Collagen Replicas in PS

Thermal (nano)imprinting in combination with a soft, elastomeric mold/“soft embossing” was used to transfer the structure of collagen micropatterns onto a PS film. Inverse replicas of the collagen structure were fabricated by first mixing a perfluoropolyether (PFPE)‐urethane dimethacrylate monomer (Fluorolink MD700, Acota) with 1‐hydroxycyclohexyl phenyl ketone photoinitiator in the ratio of 100 µL:1 µg, pouring the mixture onto the collagen micropatterns and degassing it in a vacuum chamber to remove air bubbles. The cast PFPE‐based compound was then UV‐exposed at 365 nm under N_2_ atmosphere for 30 min. Finally, PFPE replicas featuring the negative topography of the collagen fibers were peeled off the same. The PFPE replicas were then imprinted into 100 µm thick PS films (Goodfellow) at 150 °C and 10 bar for 5 min using a EITRE 6 semi‐automated nanoimprint lithography equipment (Obducat), resulting in the “positive” replication of the collagen fibers on the films. To allow cell adhesion later in the cell culture, the PS films were treated in an oxygen plasma using a PlasmaFlecto 10 plasma reactor (plasma technology) at 75 mTorr and 50 sccm O_2_ and 50 W for 30 s. In order to examine the replication fidelity of the collagen microstructure, quantification of the surface profile parameters roughness average (Ra), difference between the tallest peak and the deepest valley in the surface (Rz), and mean width of profile elements (RSm) was performed using a VK‐X250 confocal laser profilometer (KEYENCE). Samples with a size of 1.5 mm × 4 mm were punched out of the films, sterilized in 70% ethanol for 30 min and air‐dried under sterile conditions. Prior to cell culture, the samples were washed twice with Dulbecco's PBS (Sigma‐Aldrich), placed in 24‐well plates and secured at the bottom of the wells using sterilized O‐rings (ERIKS).

### Tenocyte and hMSC Isolation, Expansion, and Culture

Tendon tissues were harvested post‐mortem from 23 weeks old Cyp1a2ren strain rats. Rats were collected after euthanization considering their surplus status from the breeding program. The (“aanvraag projectvergunning dierproeven”) number of the ethical approval of the experiment in which the rats were used is AVD107002017924. Isolation of tenocytes was performed as described previously.^[^ ^42^ ^]^ The isolated tenocytes were cultured in 25 cm^2^ flasks in low‐glucose Dulbecco's Modified Eagle's Medium (DMEM; Merck) supplemented with 10% v/v fetal bovine serum (FBS; Merck) and 100 U mL^−1^ penicillin/streptomycin (Thermo Fisher Scientific), and incubated at 37 °C and 5% CO_2_ in a humidified atmosphere. Medium was changed every 2–3 days. Cells were passaged when reaching 80% confluence. Cells of passage 5 were seeded at a density of 5000 cells cm^−2^ in the low‐glucose DMEM‐based medium on the different fibrous and non‐fibrillar collagen micropatterns, replicas of the collagen micropatterns in PS, and TCP, which served as control.

hMSCs were isolated as previously described from a single donor who had given consent,^[^ ^49^ ^]^ based on a corresponding document from the Medical Ethical Committee (Medisch Ethische Toetsingscommissie; METC) of the Medisch Spectrum Twente hospital, Enschede, The Netherlands (study protocol “Functioneel weefselherstel met behulp vanuit beenmerg verkregen stamcellen;” K06‐002). Cells of passage 4 were seeded on the collagen patterns at a density of 5000 cells cm^−2^ in basic medium consisting of α‐Minimum Essential Medium (Thermo Fisher Scientific, Gibco) supplemented with 10% FBS (Sigma‐Aldrich), 0.2 × 10^−3^ mm l‐ascorbic acid 2‐phosphate magnesium salt (Sigma‐Aldrich), and 100 U mL^−1^ penicillin and 100 µg mL^−1^ streptomycin (Thermo Fisher Scientific, Gibco), or again in this basic medium but now supplemented with 20 ng mL^−1^ TGF‐β2 (PeproTech). Medium was changed every 2–3 days.

### Immunocytochemistry and Fluorescence Imaging

Immunocytochemistry was performed to assess cell morphology, and the expression of the tenogenic markers SCX after 1 and 3 days^[^ ^10^, ^45^, ^50^ ^]^ and TNC after 5 days of culture^[^ ^51^ ^]^ and of the FA protein vinculin after 3 days of culture. Following cell culture, the samples were washed with PBS and fixed with 4% w/v paraformaldehyde (Sigma‐Aldrich) at RT for 15 min. After washing the samples three times with PBS, cells were permeabilized with 0.01% v/v Triton X‐100 (Acros Organics) and blocked with goat serum (Sigma‐Aldrich; 1:100) in PBT (PBS and 0.02% Triton X‐100 with 0.5% BSA) or with 5% BSA at RT for 1 h. Next, the samples were incubated with a primary anti‐SCX antibody (Abcam; ab58655; 1:200 in 1% goat serum) at RT for 1 h or with a primary anti‐TNC antibody (Thermo Fisher Scientific; 4C8MS; 1:100 in 1% goat serum) or a primary anti‐vinculin antibody (Abcam; ab196579; 1:200 in 5% BSA) in each case at 4 °C overnight. The samples were washed three times with PBS and then incubated with a goat‐anti‐rabbit secondary antibody conjugated to Alexa Fluor 647 (Thermo Fisher Scientific; 1:500), together with phalloidin conjugated to Alexa Fluor 568 (Thermo Fisher Scientific; 1:500) in PBT for 1 h. After washing the samples three times with PBS, nuclei were labelled with DAPI (4′,6‐diamidino‐2‐phenylindole; Thermo Fisher Scientific; 1:100). The samples were then mounted with a microscopy cover slip on top using Dako Fluorescence Mounting Medium (Agilent). Fluorescence imaging was performed using a fully automated Eclipse Ti‐U inverted microscope (Nikon) combined with a Zyla 5.5 4MP camera (Andor) and a 20× or 40× objective lens.

### Image Analysis

To assess the effect of collagen microstructure on cell morphology, microscopy images were analyzed by semi‐automated image analysis using CellProfiler (https://cellprofiler.org/; version 4.2.0).^[^ ^52^ ^]^ All immunofluorescence images were acquired with the same orientation of the samples. For each of seven samples, two regions of interest (ROIs) within the area previously covered by the flow chamber were defined, namely one ROI inside and one outside the previous micropillar area. For each ROI, per sample, three images were acquired.

Custom‐made pipelines in CellProfiler were used to measure cell descriptors of shape or intensity. Briefly, nucleus morphology was identified as a primary object using the Otsu adaptive thresholding method on the DAPI channel, and cell morphology was determined using the propagation algorithm in combination with the Otsu adaptive thresholding method on the phalloidin channel. Cells touching the edges of the images were excluded from the dataset. After background correction, SCX intensity values of each pixel inside the segmented nuclear area were used to calculate the integrated SCX value of individual nuclei. This value was then normalized by the object area in pixels. TNC intensity values were calculated as mean pixel intensity in the segmented cytoplasm area.

The length of vinculin‐containing FAs was calculated using the “Analyze Particles” functionality in ImageJ. This calculation was performed converting the image into an 8‐bit rendering, and by adjusting the threshold setting outside the 95% pixel intensity distribution profile to help in eliminating the background signal.

### RT‐qPCR

Rat tenocytes were cultured for 1 or 3 days on the different collagen micropatterns, their PS replicas and the TCP control, before total RNA was isolated using the RNeasy Mini Kit (QIAGEN) according to manufacturer's protocol. Next, RNA purity and concentration was measured using a BioDrop µLite (BioDrop).

Complementary DNA (cDNA) was synthesized with the iScript cDNA synthesis kit (Bio‐Rad) starting from 200 ng of RNA for each sample following the manufacturer's instructions. Gene expression analysis was performed by RT‐qPCR using the iQ SYBR Green Supermix (Bio‐Rad) in a CFX96 Real‐Time PCR Detection Kit (Bio‐Rad) amplifying 20 ng of cDNA. Quantification of mRNA levels of tenogenic biomarkers was performed through the ΔΔCt method using glyceraldehyde 3‐phosphate dehydrogenase (GAPDH) as housekeeping gene.^[^ ^9^ ^]^ Results are presented as relative gene expression levels normalized to that of primary tenocytes cultured on the TCP controls at the same time point (1 or 3 days). Primer sequences for each marker are listed in Table S3, Supporting Information.

### Statistical Analysis

Statistical analyses were performed using Prism (GraphPad Software; version 8.4.2). Differences in quantitative immunohistochemistry results between the experimental groups and the TCP‐cultured control group were analyzed by one‐way ANOVA with a post‐hoc Tuckey HSD test. For RT‐qPCR experiments, an unpaired Student's *t*‐test after log transformation of the expression fold changes was used. A *p*‐value less than 0.05 was considered statistically significant. PCA was performed using the XLSTAT data analysis add‐on in Excel (Microsoft; version 16.18) and by correlation methods using the Pearson correlation coefficient. All quantitative data represented in this study are based on *N* = 3 independent samples.

## Conflict of Interest

R.T. and S.G. are founders, shareholders, and managing directors of the company 300MICRONS GmbH, active in the field of 3D cell culture solutions.

## Author Contributions

F.G.: Formal analysis, Investigation, Methodology, Validation, Visualization, Writing—original draft, Writing—review and editing. D.B.B.: Investigation, Writing—review and editing. H.S.R.: Investigation, Writing—review and editing. Z.T.B.: Methodology, Supervision, Validation, Writing—review and editing. C.v.B.: Funding acquisition, Writing—review and editing. S.G.: Conceptualization, Funding acquisition, Methodology, Supervision, Writing—review and editing. R.T.: Conceptualization, Funding acquisition, Methodology, Supervision, Validation, Writing—original draft, Writing—review and editing. P.H.: Conceptualization, Funding acquisition, Methodology, Supervision, Validation, Writing—review and editing.

## Supporting information

Supporting Information

## Data Availability

The data that support the findings of this study are available from the corresponding author upon reasonable request.

## References

[1] M. Franchi , A. Trirè , M. Quaranta , E. Orsini , V. Ottani , Sci. World J. 2007, 7, 404. 10.1100/tsw.2007.92 PMC5901217 17450305

[2] M. Benjamin , E. Kaiser , S. Milz , J. Anat. 2008, 212, 211. 18304204 10.1111/j.1469-7580.2008.00864.x PMC2408985

[3] J. H.‐C. Wang , J. Biomech. 2006, 39, 1563. 16000201 10.1016/j.jbiomech.2005.05.011

[4] A. Dede Eren , A. Vasilevich , E. D. Eren , P. Sudarsanam , U. Tuvshindorj , J. De Boer , J. Foolen , J. de Boer , J. Foolen , Tissue Eng., Part A 2021, 27, 1023. 33045937 10.1089/ten.TEA.2020.0249

[5] a) M. R. Citeroni , M. C. Ciardulli , V. Russo , G. Della Porta , A. Mauro , M. El Khatib , M. Di Mattia , D. Galesso , C. Barbera , N. R. Forsyth , N. Maffulli , B. Barboni , Int. J. Mol. Sci. 2020, 21, 6726; 32937830 10.3390/ijms21186726 PMC7555358

[6] a) J. Pingel , Y. Lu , T. Starborg , U. Fredberg , H. Langberg , A. Nedergaard , M. Weis , D. Eyre , M. Kjaer , K. E. Kadler , J. Anat. 2014, 224, 548; 24571576 10.1111/joa.12164 PMC3981497

[7] G. Yang , B. B. Rothrauff , R. S. Tuan , Birth Defects Res., Part C 2013, 99, 203. 10.1002/bdrc.21041 PMC4041869 24078497

[8] a) M. Silva , F. N. Ferreira , N. M. Alves , M. C. Paiva , J. Nanobiotechnol. 2020, 18, 23; 10.1186/s12951-019-0556-1 PMC6993465 32000800

[9] S. Vermeulen , A. Vasilevich , D. Tsiapalis , N. Roumans , P. Vroemen , N. R. M. Beijer , A. Dede Eren , D. Zeugolis , J. de Boer , Acta Biomater. 2019, 83, 277. 30394345 10.1016/j.actbio.2018.10.041

[10] a) A. D. Mazzocca , D. Chowaniec , M. B. Mccarthy , K. Beitzel , M. P. Cote , W. Mckinnon , R. Arciero , Knee Surg. Sports Traumatol. Arthrosc. 2012, 20, 1666; 22005966 10.1007/s00167-011-1711-x

[11] K. Von Der Mark , V. Gauss , H. Von Der Mark , P. Müller , Nature 1977, 9, 531. 10.1038/267531a0 559947

[12] A. Kapoor , E. H. G. Caporali , P. J. A. Kenis , M. C. Stewart , Acta Biomater. 2010, 6, 2580. 20045087 10.1016/j.actbio.2009.12.047

[13] A. English , A. Azeem , K. Spanoudes , E. Jones , B. Tripathi , N. Basu , K. Mcnamara , S. A. M. Tofail , N. Rooney , G. Riley , A. O'riordan , G. Cross , D. Hutmacher , M. Biggs , A. Pandit , D. I. Zeugolis , Acta Biomater. 2015, 27, 3. 26318365 10.1016/j.actbio.2015.08.035

[14] J.i Zhu , J. Li , B. Wang , W. J. Zhang , G. Zhou , Y. Cao , W. Liu , Biomaterials 2010, 31, 6952. 20638974 10.1016/j.biomaterials.2010.05.058

[15] V. Kishore , W. Bullock , X. Sun , W. S. Van Dyke , O. Akkus , Biomaterials 2012, 33, 2137. 22177622 10.1016/j.biomaterials.2011.11.066 PMC3279298

[16] M. Younesi , A. Islam , V. Kishore , S. Panit , O. Akkus , Biofabrication 2015, 7, 035001. 26069162 10.1088/1758-5090/7/3/035001 PMC4489851

[17] a) S. Chen , N. Hirota , M. Okuda , M. Takeguchi , H. Kobayashi , N. Hanagata , T. Ikoma , Acta Biomater. 2011, 7, 644; 20851220 10.1016/j.actbio.2010.09.014

[18] A. Sensini , L. Cristofolini , A. Zucchelli , M. L. Focarete , C. Gualandi , A. De Mori , A. P. Kao , M. Roldo , G. Blunn , G. Tozzi , J. Microsc. 2020, 277, 160. 31339556 10.1111/jmi.12827

[19] G. M. Whitesides , Nature 2006, 442, 368. 16871203 10.1038/nature05058

[20] S. Köster , H. M. Evans , J. Y. Wong , T. Pfohl , Biomacromolecules 2008, 9, 199. 18078321 10.1021/bm700973t PMC2673469

[21] P. Lee , R. Lin , J. Moon , L. P. Lee , Biomed. Microdevices 2006, 8, 35. 16491329 10.1007/s10544-006-6380-z

[22] P. Kivanany , K. Grose , N. Yonet‐Tanyeri , S. Manohar , Y. Sunkara , K. Lam , D. Schmidtke , V. Varner , W. Petroll , J. Funct. Biomater. 2018, 9, 54. 30248890 10.3390/jfb9040054 PMC6306816

[23] B. Lanfer , A. Hermann , M. Kirsch , U. Freudenberg , U. Reuner , C. Werner , A. Storch , Tissue Eng., Part A 2010, 16, 1103. 19860550 10.1089/ten.TEA.2009.0282

[24] B. Lanfer , F. P. Seib , U. Freudenberg , D. Stamov , T. Bley , M. Bornhäuser , C. Werner , Biomaterials 2009, 30, 5950. 19674785 10.1016/j.biomaterials.2009.07.039

[25] R. Gottardi , U. Hansen , R. Raiteri , M. Loparic , M. Düggelin , D. Mathys , N. F. Friederich , P. Bruckner , M. Stolz , PLoS One 2016, 11, e0163552. 27780246 10.1371/journal.pone.0163552 PMC5079628

[26] A. Dede Eren , E. D. Eren , T. J. S. Wilting , J. de Boer , H. Gelderblom , J. Foolen , Sci. Rep. 2021, 11, 1516. 33452334 10.1038/s41598-021-81054-5 PMC7810981

[27] P. Alberton , C. Popov , M. Prägert , J. Kohler , C. Shukunami , M. Schieker , D. Docheva , Stem Cells Dev. 2012, 21, 846. 21988170 10.1089/scd.2011.0150 PMC3315756

[28] E. Chen , L. Yang , C. Ye , W. Zhang , J. Ran , D. Xue , Z. Wang , Z. Pan , Q. Hu , Acta Biomater. 2018, 73, 377. 29678676 10.1016/j.actbio.2018.04.027

[29] Z.i Yin , X. Chen , J. L. Chen , W. L. Shen , T. M. Hieu Nguyen , L. Gao , H. W. Ouyang , Biomaterials 2010, 31, 2163. 19995669 10.1016/j.biomaterials.2009.11.083

[30] T. Peng , L. Liu , A. L. Maclean , C. W. Wong , W. Zhao , Q. Nie , BMC Syst. Biol. 2017, 11, 55. 28511648 10.1186/s12918-017-0429-x PMC5434622

[31] E. Maharam , M. Yaport , N. L. Villanueva , T. Akinyibi , D. Laudier , Z. He , D. J. Leong , H. B. Sun , Bone Res. 2015, 3, 15015. 26509098 10.1038/boneres.2015.15 PMC4605238

[32] C. Luxenburg , R. Zaidel‐Bar , Exp. Cell Res. 2019, 378, 232. 30872138 10.1016/j.yexcr.2019.03.016

[33] S. K. Theodossiou , J. B. Murray , L. A. Hold , J. M. Courtright , A. M. Carper , N. R. Schiele , Stem Cell Res. Ther. 2021, 12, 88. 33499914 10.1186/s13287-021-02167-2 PMC7836508

[34] a) N. R. M. Beijer , Z. M. Nauryzgaliyeva , E. M. Arteaga , L. Pieuchot , K. Anselme , J. Van De Peppel , A. S. Vasilevich , N. Groen , N. Roumans , D. G. A. J. Hebels , J. D. Boer , Sci. Rep. 2019, 9, 9099; 31235713 10.1038/s41598-019-45284-y PMC6591423

[35] A. D. Schoenenberger , J. Foolen , P. Moor , U. Silvan , J. G. Snedeker , Acta Biomater. 2018, 71, 306. 29530822 10.1016/j.actbio.2018.03.004

[36] D. Wang , C. C. M. Pun , S. Huang , T. C. M. Tang , K. K. W. Ho , B. B. Rothrauff , P. S. H. Yung , A. M. Blocki , E. D. F. Ker , R. S. Tuan , FASEB J. 2020, 34, 8172. 32301551 10.1096/fj.201902377RR

[37] E. Berthet , C. Chen , K. Butcher , R. A. Schneider , T. Alliston , M. Amirtharajah , J. Orthop. Res. 2013, 31, 1475. 23653374 10.1002/jor.22382 PMC3960924

[38] C. González‐García , S. R. Sousa , D. Moratal , P. Rico , M. Salmerón‐Sánchez , Colloids Surf., B 2010, 77, 181. 10.1016/j.colsurfb.2010.01.021 20185279

[39] P.‐O. Bagnaninchi , Y. Yang , A. J. El Haj , N. Maffulli , U. Bosch , Br. J. Sports Med. 2007, 41, e10; 17062654 10.1136/bjsm.2006.030643 PMC2465448

[40] C. Lehner , G. Spitzer , R. Gehwolf , A. Wagner , N. Weissenbacher , C. Deininger , K. Emmanuel , F. Wichlas , H. Tempfer , A. Traweger , Dis. Models Mech. 2019, 12, dmm041384. 10.1242/dmm.041384 PMC6918766 31744815

[41] Y. Xu , H. Yin , J. Chu , D. Eglin , T. Serra , D. Docheva , Biomater. Sci. 2021, 9, 1237. 33576754 10.1039/d0bm01127d

[42] W.‐C. Tsai , J.‐H. S. Pang , C.‐C. Hsu , N.‐K. Chu , M.‐S. Lin , C.‐F. Hu , J. Orthop. Res. 2006, 24, 1310. 16705693 10.1002/jor.20130

[43] B. Xu , G. Song , Y. Ju , X. Li , Y. Song , S. Watanabe , J. Cell. Physiol. 2012, 227, 2722. 21898412 10.1002/jcp.23016

[44] A. Dede Eren , A. W. A. Lucassen , U. Tuvshindorj , R. Truckenmüller , S. Giselbrecht , E. D. Eren , M. O. Tas , P. Sudarsanam , J. De Boer , Front. Cell Dev. Biol. 2022, 10, 863721. 35721512 10.3389/fcell.2022.863721 PMC9203963

[45] Y. Komiyama , S. Ohba , N. Shimohata , K. Nakajima , H. Hojo , F. Yano , T. Takato , D. Docheva , C. Shukunami , Y. Hiraki , U.‐I. Chung , PLoS One 2013, 8, e60203. 23593173 10.1371/journal.pone.0060203 PMC3622668

[46] a) L. Sun , D. Pereira , Q. Wang , D. B. Barata , R. Truckenmüller , Z. Li , X. Xu , P. Habibovic , PLoS One 2016, 11, e0161466; 27571520 10.1371/journal.pone.0161466 PMC5003369

[47] A. E. C. Nichols , R. E. Settlage , S. R. Werre , L. A. Dahlgren , BMC Cell Biol. 2018, 19, 14. 30086712 10.1186/s12860-018-0166-z PMC6081934

[48] S. Testa , M. Costantini , E. Fornetti , S. Bernardini , M. Trombetta , D. Seliktar , S. Cannata , A. Rainer , C. Gargioli , J. Cell. Mol. Med. 2017, 21, 2711. 28470843 10.1111/jcmm.13186 PMC5661263

[49] S. K. Both , A. J. C. V. D. Muijsenberg , C. A. V. Blitterswijk , J. D.e Boer , J. D. D.e Bruijn , Tissue Eng. 2007, 13, 3. 17518576 10.1089/ten.2005.0513

[50] a) F. Busch , A. Mobasheri , P. Shayan , C. Lueders , R. Stahlmann , M. Shakibaei , J. Biol. Chem. 2012, 287, 38050; 22936809 10.1074/jbc.M112.377028 PMC3488075

[51] C. Milet , D. Duprez , Ann. Transl. Med. 2015, 3, S33. 26046080 10.3978/j.issn.2305-5839.2015.03.64 PMC4437935

[52] A. E. Carpenter , T. R. Jones , M. R. Lamprecht , C. Clarke , I.n Kang , O. Friman , D. A. Guertin , J. Chang , R. A. Lindquist , J. Moffat , P. Golland , D. M. Sabatini , Genome Biol. 2006, 7, R100. 17076895 10.1186/gb-2006-7-10-r100 PMC1794559

