# Supplementary material for: Microfluidically Aligned Collagen to Maintain the Phenotype of Tenocytes In Vitro
Source: Adv Healthc Mater. 2023 Dec 8;13(6):2303672. doi: 10.1002/adhm.202303672 (PMC11468977; doi:10.1002/adhm.202303672)
Supplement: Supplementary file 1 — Supporting Information [file ADHM-13-2303672-s001.pdf]

ADVANCED HEALTHCARE MATERIALS

Supporting Information

for *Adv. Healthcare Mater.*, DOI 10.1002/adhm.202303672

Microfluidically Aligned Collagen to Maintain the Phenotype of Tenocytes In Vitro

Francesca Giacomini, David Baião Barata, Hoon Suk Rho, Zeinab Tahmasebi Birgani, Clemens van Blitterswijk, Stefan Giselbrecht, Roman Truckenmüller and Pamela Habibović*

Supporting Information

Microfluidically Aligned Collagen to Maintain the Phenotype of Tenocytes *in Vitro*

Francesca Giacomini, David Baião Barata, Hoon Suk Rho, Zeinab Tahmasebi Birgani, Clemens van Blitterswijk, Stefan Giselbrecht†, Roman Truckenmüller†, Pamela Habibović†**

†these authors equally contributed to this work

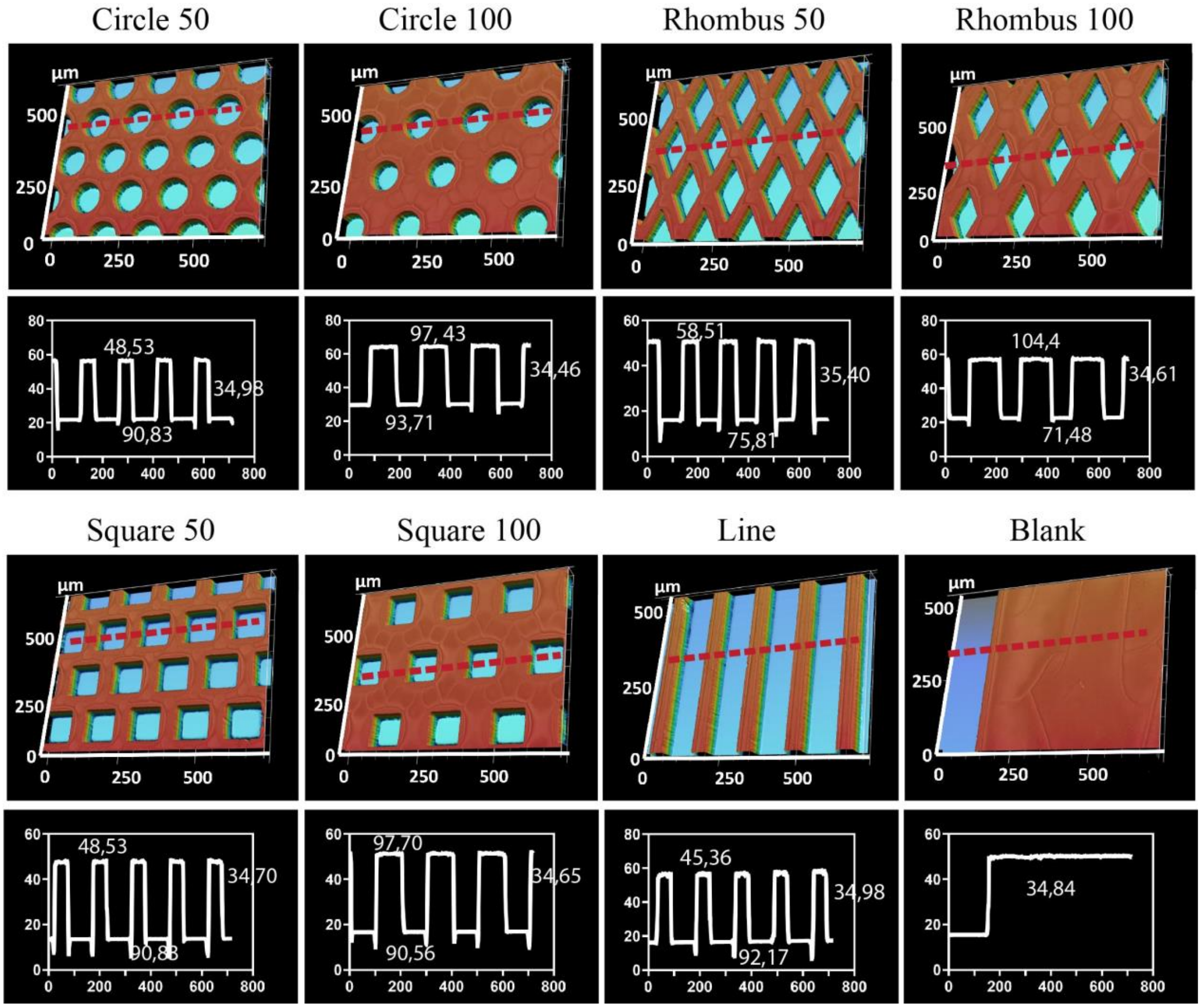

Figure S1.
Dimensional characterization of the casting mold. Results of confocal laser profilometry scans visualizing and dimensionally characterizing the SU-8 lithography structures of the molds. Spacing between the microstructures, and their width and depth, the latter resulting in the height of the microfluidic chambers, are indicated. Red dashed lines indicate the paths chosen for the characterization of the height profiles.

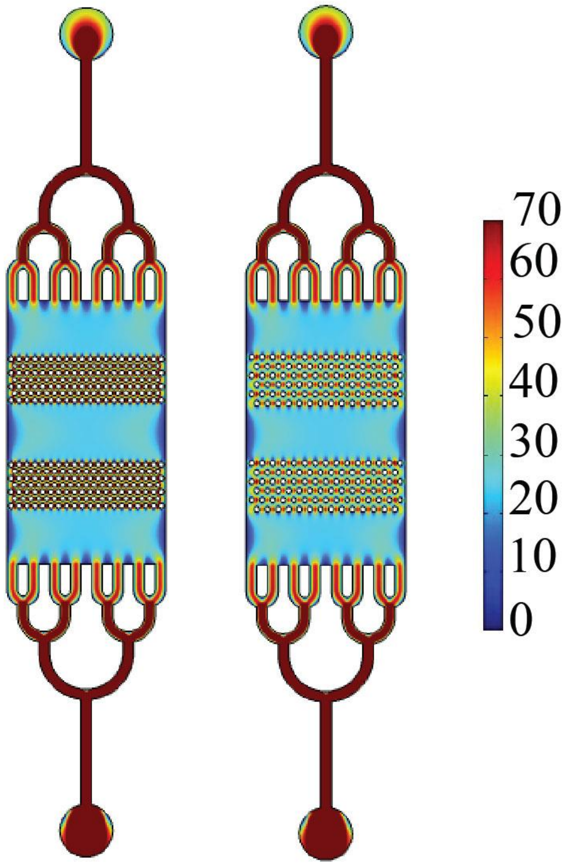

Figure S2.
CFD simulations of the flow fields in the micropillared microfluidic chambers. Velocity heat maps displaying the laminar flow velocities from simulations of the laminar flow through the microfluidic platforms for the micropillar array designs Circle 50 (left) and Circle 100 (right). The color legend at the right represents the local flow velocities in mm/s.

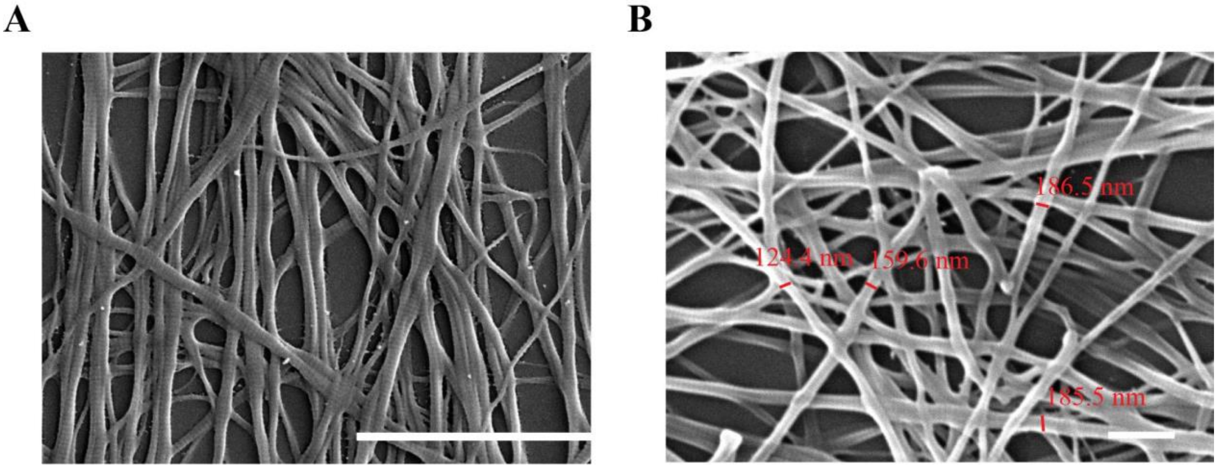

Figure S3.

Collagen fiber characterization. A) High-magnification SEM image of collagen fibril bundles produced using the microfluidic platform show the characteristic 67 nm-banding pattern. Scale bar represents 4 µm. B) Quantification of the diameters of the collagen fibrils on an SEM image. Scale bar represents 1 µm.

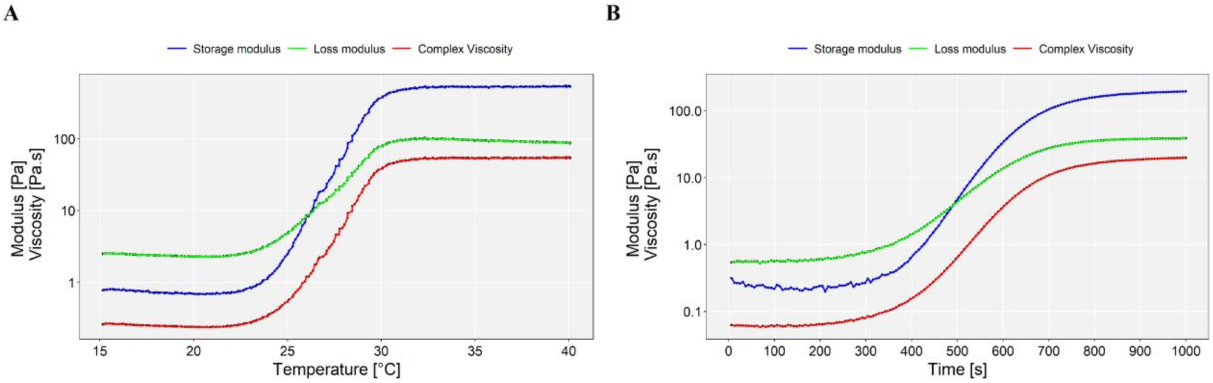

Figure S4.

Rheological characterization of the collagen solution (and hydrogel). A,B) Shear storage (blue) and loss modulus (green) and complex velocity (red) of a 1.5 mg/ml collagen solution exposed to a temperature ramp of 1 K/min between 15 and 37 ˚C, measured on a Discovery Hybrid Rheometer (DHR)-2 (TA Instruments), and displayed as a function of temperature (A) and time (B).

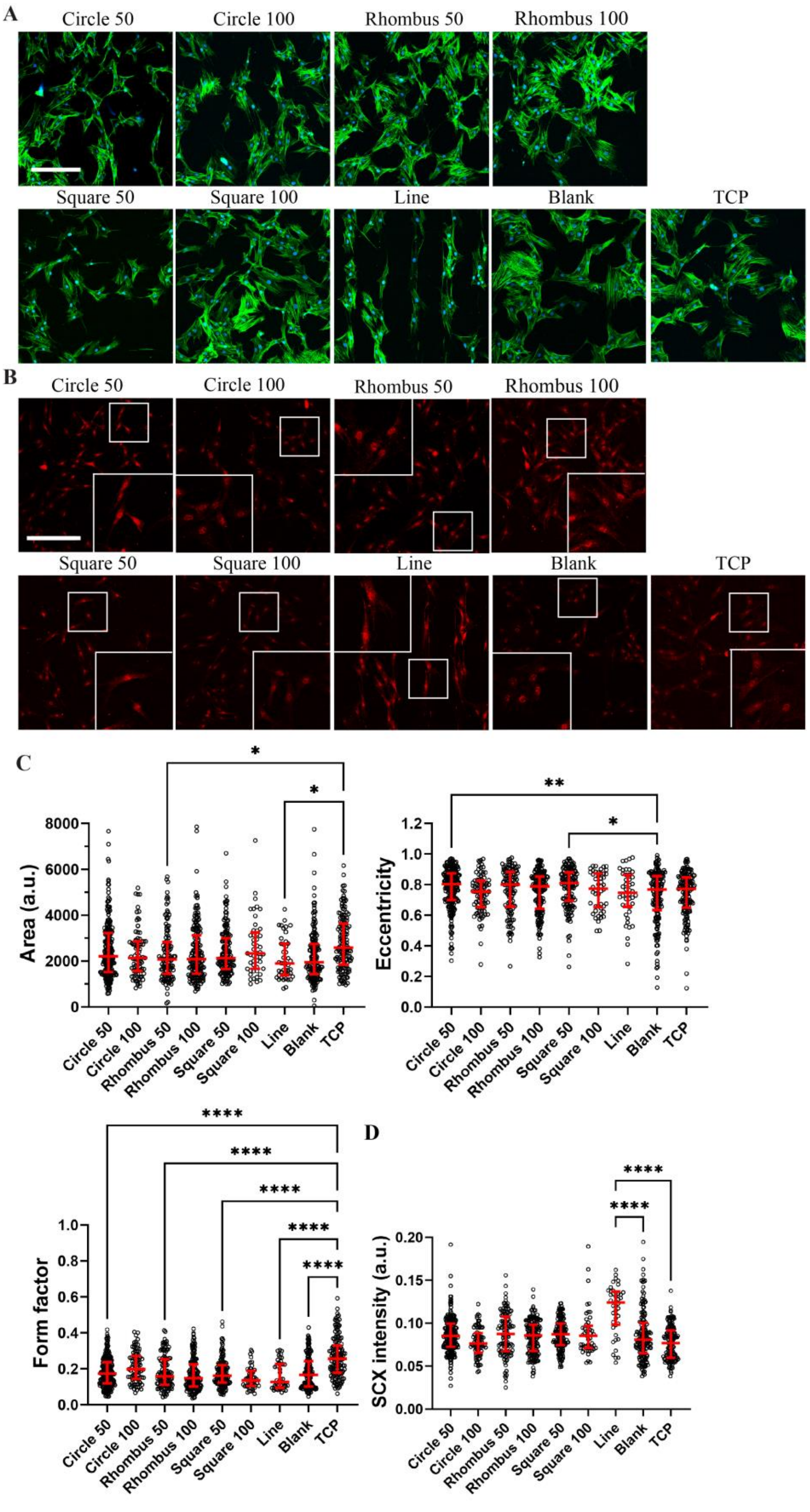

Figure S5.

Characterization of tenocytes cultured on collagen fibers for 1 day. A,B) Representative immunocytochemistry images of rat tenocytes cultured on fibrous collagen formed in

microfluidic devices with different micropillar arrays and without (Blank) and on TCP. Tenocytes were stained with DAPI to visualize cell nuclei (blue; A) and phalloidin to visualize F-actin (green; A), and for SCX (red; B). Scale bars represent 50 µm and apply to all images in the same column. Insets in the corners of the images show enlarged views of the areas delineated by the white boxes. C) Quantitative analysis of cell shape descriptors, including cell area (top left), eccentricity (top right) and form factor (bottom left). Median values are represented by horizontal lines and interquartile ranges by error bars. **p* < 0.05, ***p* < 0.01, *****p* < 0.0001. *N* = 3. D) Quantitative analysis of nuclear SCX intensity. Median values are represented by horizontal lines and interquartile ranges by error bars. *****p* < 0.0001. *N* = 3.

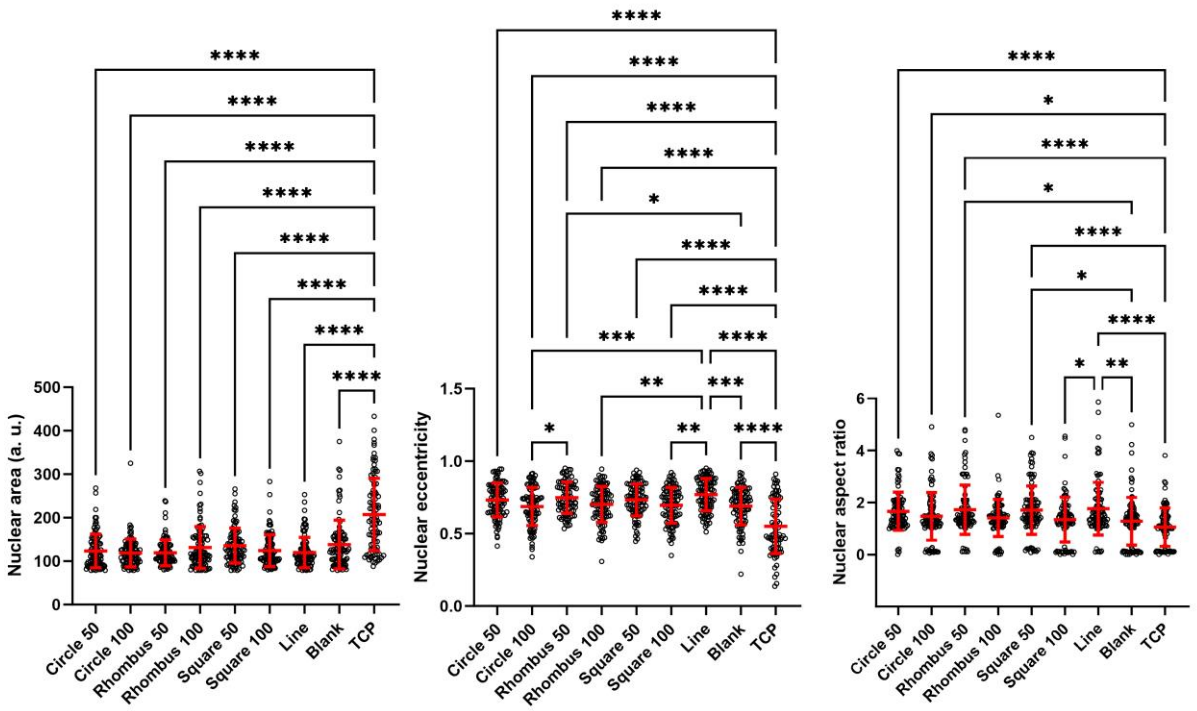

Figure S6.

Morphological characterization of the nuclei of tenocytes cultured on collagen fibers for 3 days. Quantitative analysis of nuclear area (left), eccentricity (middle) and aspect ratio (right). Median values are represented by horizontal lines and interquartile ranges by error bars. **p* < 0.05, ***p* < 0.01, ****p* < 0.001, *****p* < 0.0001. *N* = 3.

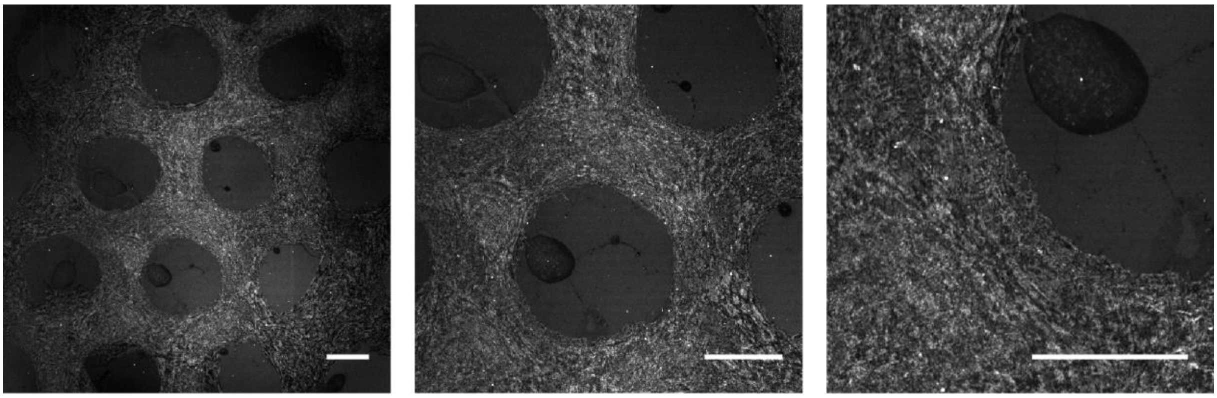

Figure S7.
Circle 50 collagen micropatterns with cells after 5 days of culture. Label-free fluorescence microscopy images (original image and zoomed-in ones; obtained using reflection mode at 488 nm) of collagen fibers. The images show that the collagen micropatterns have neither visibly degraded and/or (partly) disappeared nor detached and/or contracted. Scale bars represent 50 µm.

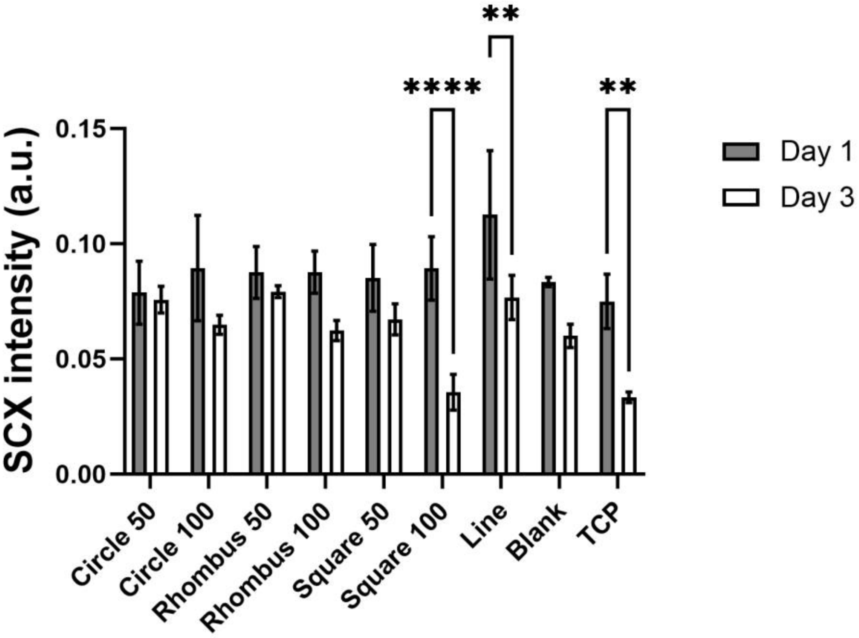

Figure S8.
Comparison of nuclear SCX intensity at day 1 and day 3. Bar chart representation of SCX expression levels of tenocytes cultured on different collagen micropatterns after 1 and 3 days. Bars and error bars represent mean values and standard deviations, respectively. $^{**}p < 0.01$, $^{****}p < 0.0001$. $N = 3$.

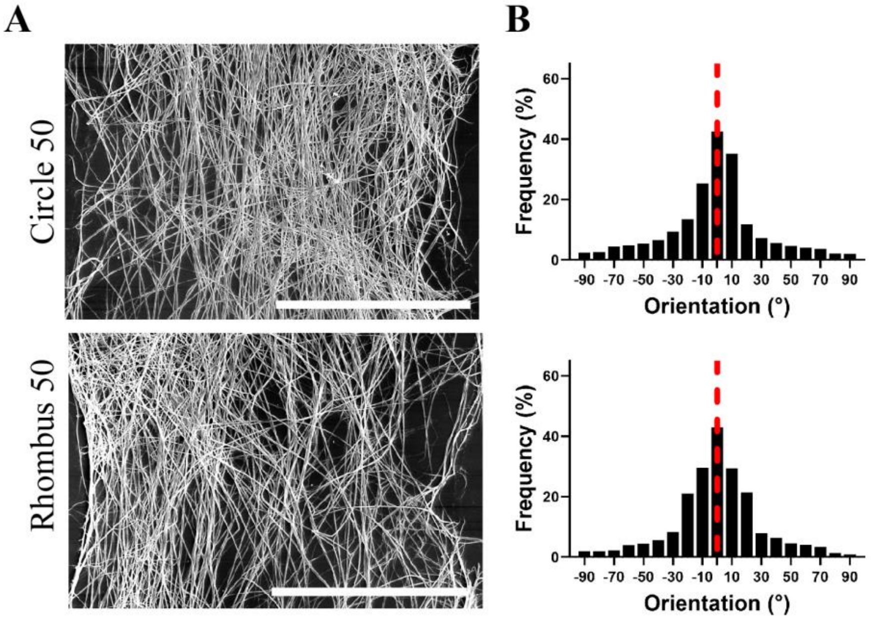

Figure S9.

Formation of collagen fibers in microfluidic devices for large-area patterning. A) SEM images of collagen fibers formed in Circle 50 (top) and Rhombus 50microfluidic devices for large-area patterning (bottom). Scale bars represent 50 µm. B) Quantitative analysis of collagen fiber alignment. Histograms represent the relative frequency (y-axis) of total fibers within each angle range versus the fiber orientation angle (x-axis), with 0° (blue dashed line) set as parallel to the left image border. *N* = 3.

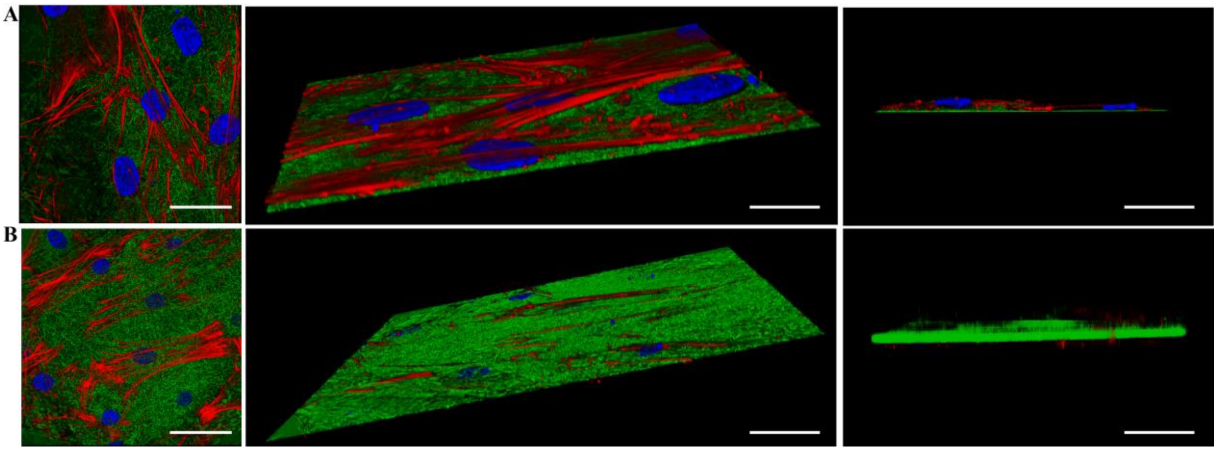

Figure S10.

3D culture established in Circle 50 device. A,B) Maximum projection (left column), 3D reconstruction (middle column) and side-view images of confocal fluorescent microscopy images (right column) of rat tenocytes cultured for 3 days on aligned collagen fibers (A) and three-dimensionally embedded in collagen fibers in the microfluidic device (B). Cells were stained with DAPI to visualize cell nuclei (blue) and phalloidin to visualize F-actin (red).

Collagen fibers were visualized using label-free fluorescence microscopy (using reflection mode at 488 nm; green). Scale bars in left, middle and right column represent 50, 20 and 50 µm, respectively.

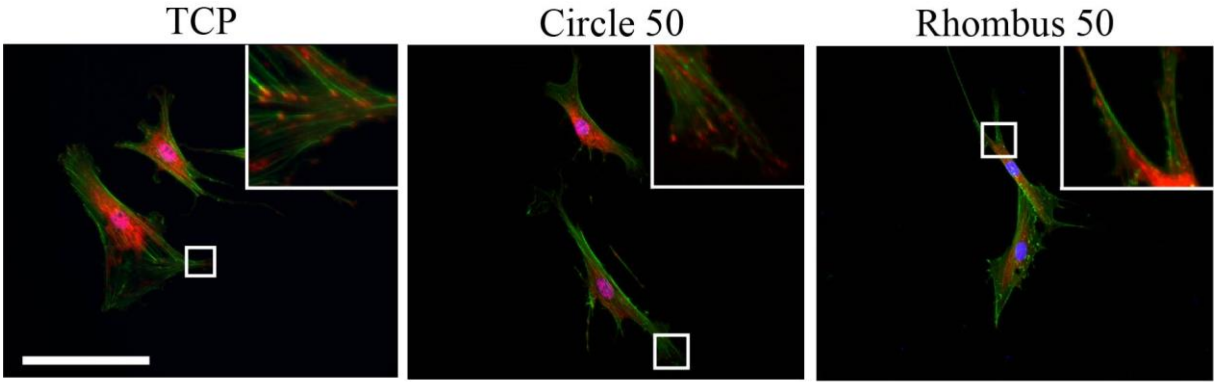

Figure S11.

FA formation in tenocytes cultured for 1 day on collagen micropatterns. Representative fluorescence microscopy images of vinculin staining (red) in tenocytes upon attachment to different substrates and culture for 1 day. Cells were stained also with DAPI to visualize cell nuclei (blue) and phalloidin to visualize F-actin (green). Scale bar represents 50 µm and applies to all images. Insets in the upper right corners of the images show enlarged views of the areas delineated by the white boxes.

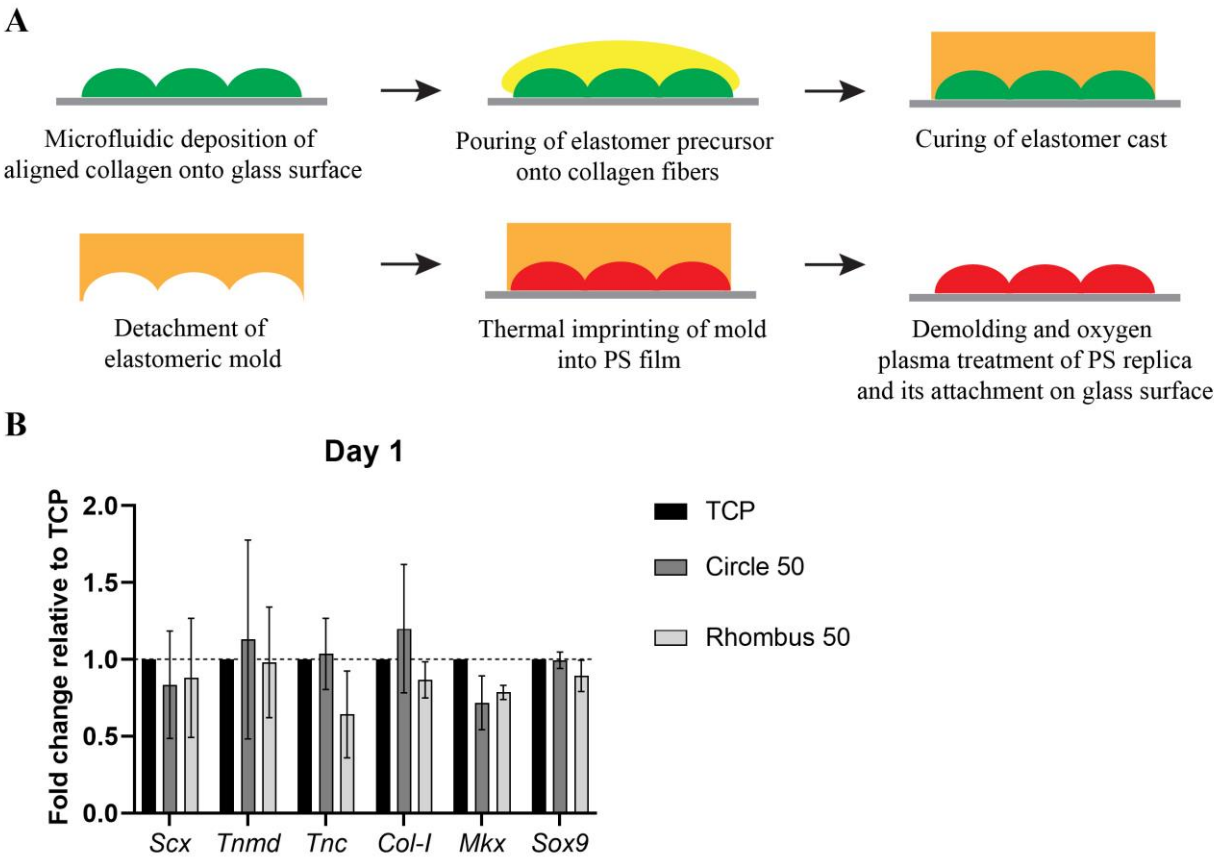

Figure S12.

Preparation and characterization of PS replicas of fibrous collagen micropatterns. A) Schematic representation of the fibers' replication into a PS film using imprinting. B) Gene expression levels of tenocytes cultured for 1 day on PS replicas of aligned collagen fibers obtained with microfluidic devices for large-area patterning. Values are given as fold changes relative to the expression in tenocytes cultured on TCP. Bars and error bars represent mean values and standard deviations, respectively. *N* = 3.

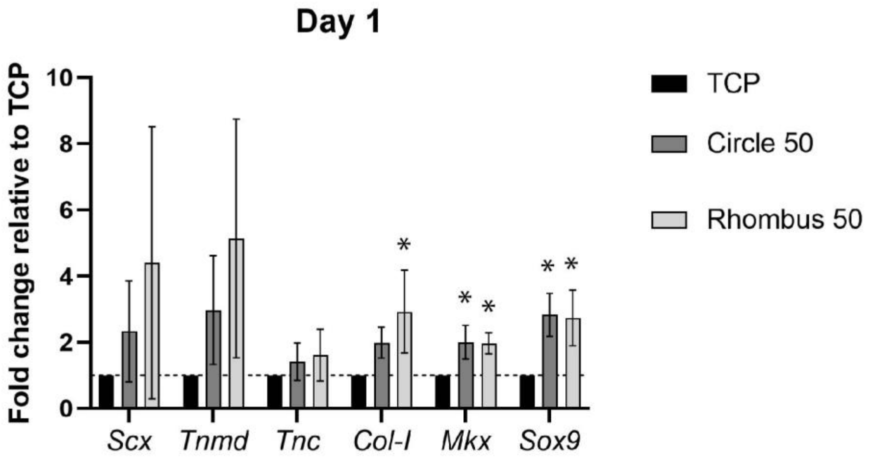

Figure S13.

Gene expression levels of tenocytes cultured on non-fibrillar collagen coated-surfaces for 1 day. Values are given as fold changes relative to the expression in tenocytes cultured on TCP. Bars and error bars represent mean values and standard deviations, respectively. *$p < 0.05$. $N = 3$.

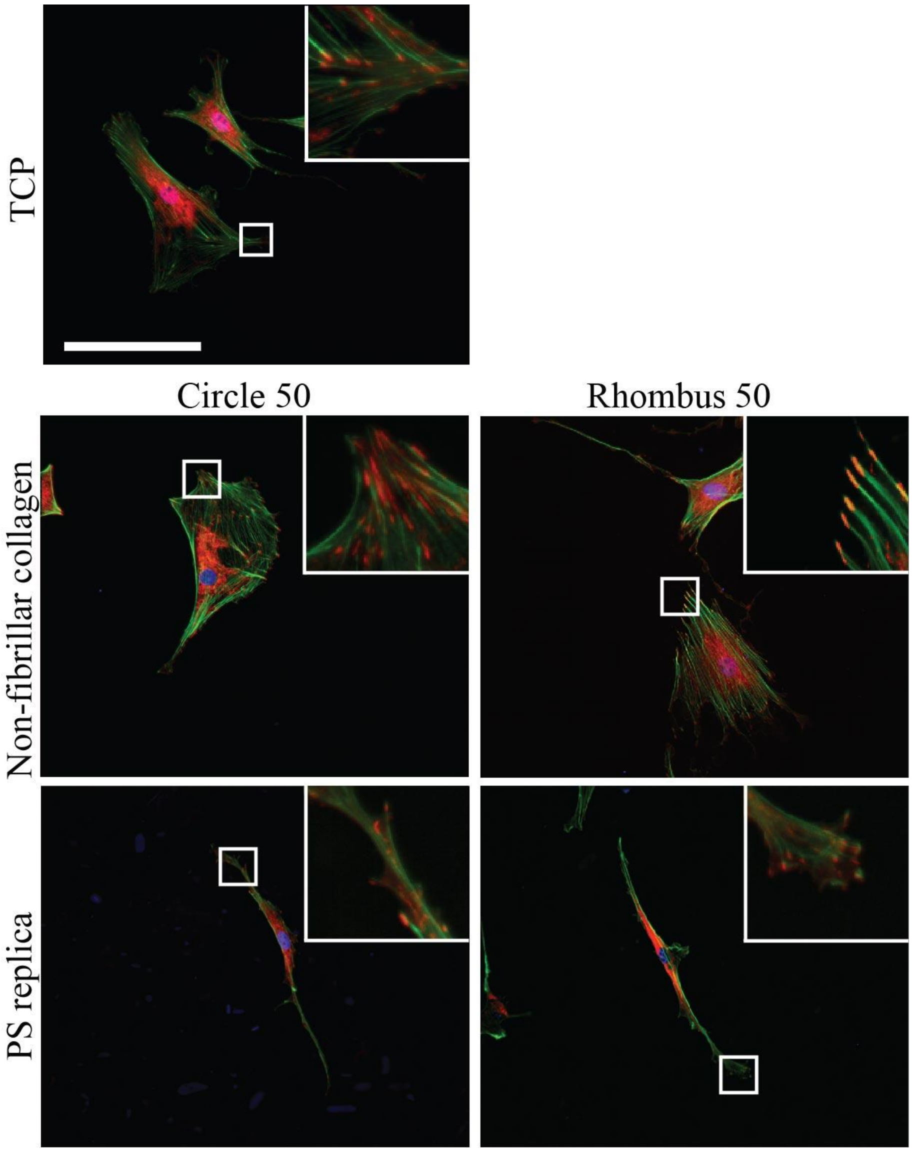

Figure S14.

Focal adhesion formation in tenocytes cultured for 1 day on non-fibrillar collagen and PS replica. Representative fluorescence microscopy images of vinculin staining (red) in tenocytes upon attachment to non-fibrillar collagen or PS replicas and culture for 1 day. Cells were also stained with DAPI to visualize cell nuclei (blue) and phalloidin to visualize F-actin (green). Scale bar represents 50 µm and applies to all images. Insets in the upper right corners of the images show enlarged views of the areas delineated by the white boxes.

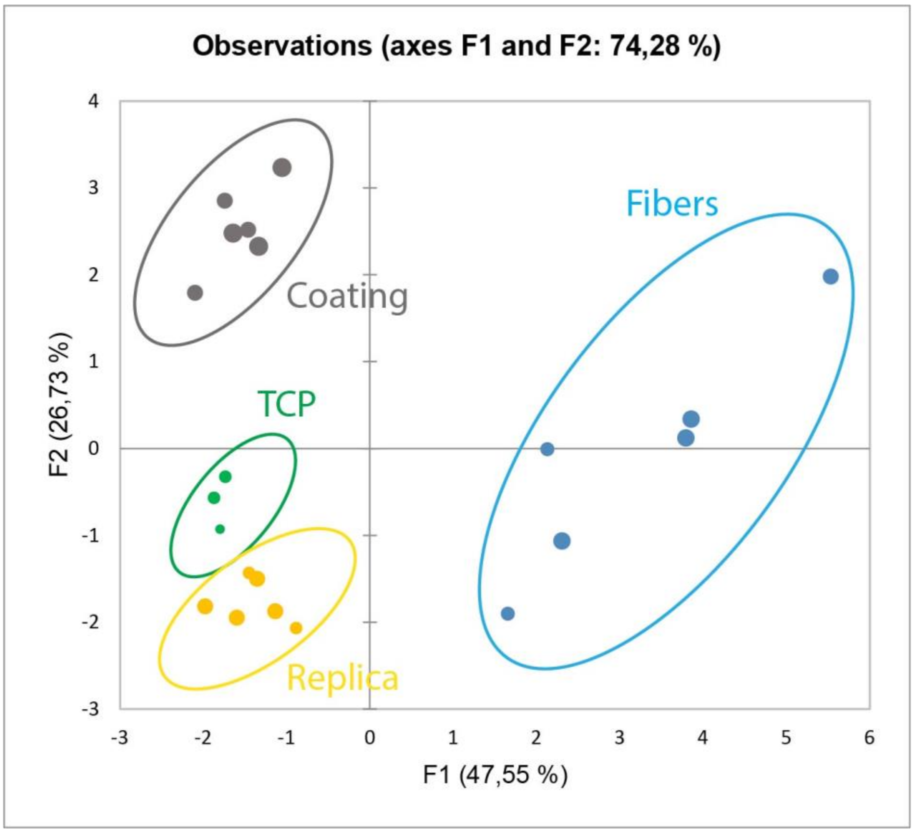

Figure S15.

PCA of cell shape descriptors, protein and gene profiles of tenocytes cultured on fibrous collagen micropatterns (‘Fibers’), non-fibrillar collagen micropatterns (‘Coating’), collagen fiber replicas (‘Replica’) in PS and TCP. PCA plot shows clustering based on similarity according to the first principal component (47.55% of total variance) and second principal component analysis (26.73% of total variance). Each dot represents a biological replicate. Ellipses were manually added to the plot to visualize clusters.

Table S1.

Dimensions of the different micropillar array designs.

Micropattern design	Distance[1] between hexagonally arranged grid points/parallel grid lines (μm)	Pillar diameter/side length/ minor × major axis length/width (μm)	Interspace[2] between pillar/ridge walls (μm)
Circle 50	150	100	50
Circle 100	200	100	100
Rhombus 50	150	100 × 200	50
Rhombus 100	200	100 × 200	100
Square 50	150	100 × 100	50
Square 100	200	100 × 100	100
Line	150	100	50

[1]Minimum distance; [2]Minimum interspace.

Table S2.

Orientation index of collagen fibers for the different micropillar array designs.

Micropattern design	Orientation index (%)
Circle 50	60 ± 3.905
Circle 100	32 ± 2.855
Rhombus 50	50 ± 1.749
Rhombus 100	31 ± 5.799
Square 50	55 ± 3.777
Square 100	38 ± 1.209
Line	50 ± 5.252
Blank	22 ± 4.866

Table S3.

Primer sequences used for RT-qPCR.

Primer name	Forward Primer	Reverse Primer
Scx	GCACCTTCTGCCTCAGCAAC	TTCTGTCACGGTCTTTGCTCA
Tnmd	CTACAGCAATGGCGAGAAGAAGAAG	GACCTACAAAGTAGATGCCAGTGTATC
Tnc	CCTGTCCCAATGACTGCAGC	GGTACTCAGTGACCCGCATC
Col-I	ATCAGCCCAAACCCCAAGGAGA	CGCAGGAAGGTCAGCTGGATAG
Mkx	ATGACTCCTGCTCTGAAGATGG	CCCCTTTAATCACAGTGTGGTG
Sox9	ATCTTCAAGGCGCTGCAA	CGGTGGACCCTGAGATTG
Gapdh	CCTGGTCACCAGGGCTGC	CGCTCCTGGAAGATGGTGATG
